# Macrophage metabolic rewiring rejuvenates muscle Raman signatures and cellular remodeling during regrowth in aged mice

**DOI:** 10.1172/jci.insight.194303

**Published:** 2025-09-09

**Authors:** Zachary J. Fennel, Negar Kosari, Paul-Emile Bourrant, Elena M. Yee, Robert J. Castro, Anu S. Kurian, Jonathan Palmer, Morgan Christensen, Katsuhiko Funai, Ryan M. O’Connell, Anhong Zhou, Micah J. Drummond

**Affiliations:** 1 Utah Center for Metabolic Health,; 2 Department of Physical Therapy and Athletic Training, and; 3 Molecular Medicine Program, University of Utah, Salt Lake City, Utah, USA.; 4 Department of Biological Engineering, Utah State University, Logan Utah, USA.; 5 Department of Nutrition & Integrative Physiology,; 6 Spencer Fox Eccles School of Medicine, and; 7 Department of Pathology, Division of Microbiology and Immunology, University of Utah, Salt Lake City, Utah, USA.

**Keywords:** Aging, Metabolism, Muscle biology, Fibrosis, Immunotherapy, Macrophages

## Abstract

Impaired muscle regrowth in aging is underpinned by reduced proinflammatory macrophage function and subsequently impaired muscle cellular remodeling. Macrophage phenotype is metabolically controlled through TCA intermediate accumulation and activation of HIF1A. We hypothesized that transient hypoxia following disuse in old mice would enhance macrophage metabolic inflammatory function, thereby improving muscle cellular remodeling and recovery. Old (20 months) and young adult mice (4 months) were exposed to acute (24 hour) normobaric hypoxia immediately following 14 days of hind limb unloading and assessed during early reambulation (4 and 7 days) compared to age-matched controls. Treated aged mice had improved proinflammatory macrophage profiles, muscle cellular remodeling, and functional muscle recovery to the levels of young control mice. Likewise, young adult mice had enhanced muscle remodeling and functional recovery when treated with acute hypoxia. Treatment in aged mice restored the muscle molecular fingerprint and biochemical spectral patterns (Raman spectroscopy) observed in young mice and strongly correlated with improved collagen remodeling. Finally, intramuscular delivery of hypoxia-treated macrophages recapitulated the muscle remodeling and recovery effects of whole-body hypoxic exposure in old mice. These results emphasize the role of proinflammatory macrophages during muscle regrowth in aging and highlight immunometabolic approaches as a route to improve muscle cellular dynamics and regrowth.

## Abstract 2

Macrophages are dysfunctional in aging and contribute to impaired muscle recovery. We show that aged macrophage function can be reversed by metabolically altering macrophage function using transient hypoxia thereby restoring muscle recovery to young levels.

## Graphical abstract

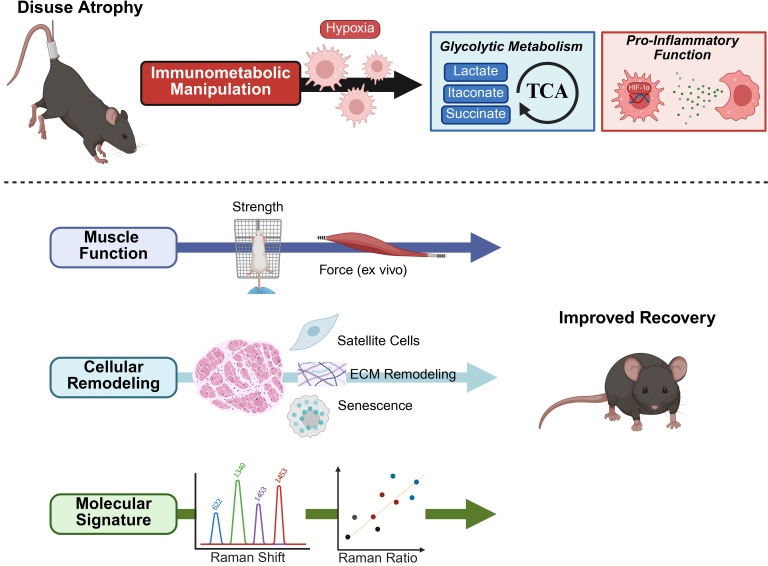

## Introduction

Skeletal muscle aging is characterized by progressive atrophy and functional decline triggered by the hallmarks of aging, including disrupted intracellular communication (1, 2). Of concern, periodic bouts of disuse, injury, or illness-related muscular atrophy can hasten muscular declines, increasing health and mortality risk (3, 4). While disuse atrophy imposes health detriments across the lifespan, the effects are heightened when aging and disuse collide, resulting in delayed or incomplete recovery (5, 6) following muscle atrophy (7–9). Therefore, the development of targeted strategies to improve muscle regrowth following disuse atrophy in aging are highly warranted.

Poor muscle remodeling in aging is underpinned by impaired cellular and molecular responses within the muscle niche that include macrophages, satellite cells, fibroblasts, and fibro/adipogenic progenitor cells (FAPs) (10, 11). Deficits in the responsiveness and function of proinflammatory-like macrophages have emerged as critical mediators of muscle regrowth in aging (12–14). Indeed, we and others have noted that proinflammatory-like macrophage polarization and function is reduced in aged muscle following reloading from disuse atrophy and coincides with delayed recovery outcomes compared with young counterparts (7, 8, 12, 15). The essential role of proinflammatory macrophages during tissue remodeling are well appreciated given that they are early responders to facilitate the clearance of tissue debris (16) and initiate intracellular communication such as stimulation of satellite cell proliferation (17) and regulation of the deleterious accumulation of collagen and intramuscular adipose from fibroblasts and FAPs (18). Therefore, approaches to manipulate proinflammatory macrophage function in aging muscle during tissue regrowth are justified.

Macrophage polarization and function are tightly controlled at the metabolic level through glycolytic, redox, and hypoxia-inducible factor-1α–related (HIF1A-related) metabolic pathways (19). Given that aging coincides with notable impairment in macrophage proinflammatory and metabolic function (8), these dysfunctional metabolic nodes may be theoretically corrected through targeted immunomodulatory manipulation (20). Therefore, in the current investigation, we leveraged whole-body and cell-specific hypoxic exposure as a tool to enhance macrophage immunometabolism and improve muscle regrowth in old mice. Furthermore, utilizing emerging techniques in Raman spectroscopy, we have characterized the molecular fingerprint and biochemical recovery pattern of aging skeletal muscle following immunometabolic intervention. Finally, we performed adoptive transfer experiments in which we intramuscularly delivered hypoxia-treated macrophages following disuse atrophy in aged mice to better explore the immunomodulatory effects in a cell-specific fashion. We hypothesized that acute hypoxic exposure in aged mice would stimulate macrophages with greater proinflammatory immunometabolic function, thus restoring cellular and molecular remodeling and functional regrowth to a youthful level. Alternatively, we hypothesized that young mice would not receive similar benefits from hypoxia treatment following disuse.

## Results

### Hypoxic exposure enhances functional recovery from disuse.

**Figure 1:**
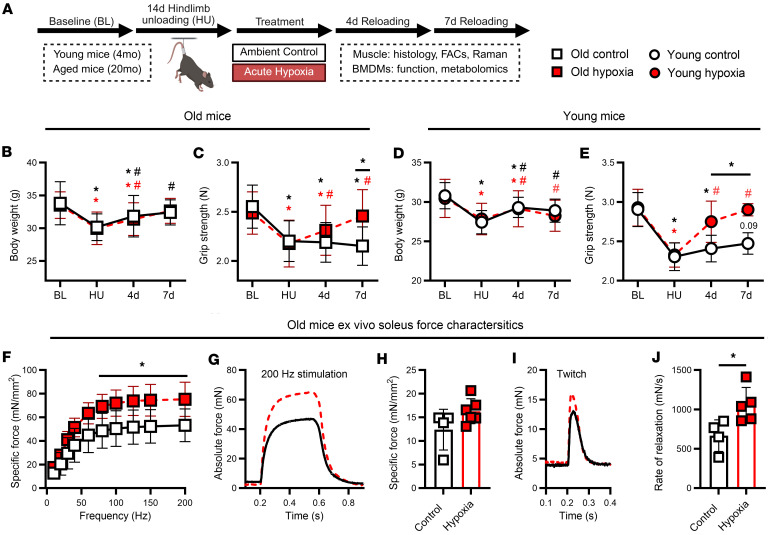
Muscle functional recovery from disuse. (**A**) Summary of experimental design. (**B**–**E**) Body weight and grip strength (*n* = 6–20) at baseline (BL), following hind limb unloading (HU), and 4 and 7 days of recovery from HU in old and young mice. (**F** and **G**) Ex vivo soleus specific force frequency (mN/mm^2^) from 10 to 200 Hz (*n* = 4–5) and representative absolute force tracing (mN) at 200 Hz stimulation from old mice. (**H** and **I**) Soleus-specific twitch at optimal length and representative peak twitch tracing. (**J**) Soleus average rate of relaxation (mN/s) between 20% and 80% of force frequency analysis. In **B**–**F**, data points represent mean of individual mice; in **H** and **J**, each data point represents an individual mouse. Data presented as mean ± SD. *P < 0.05 for difference from BL or between groups; an asterisk (*) over a solid line in **B**–**E** indicates a difference at or between those time points. Red color indicates hypoxia exposure, white color indicates control. ^#^*P* < 0.05 for difference from HU. Significance assessed by mixed effects ANOVA (**B** and **C**), 2-way ANOVA with Holm-Bonferroni test (**D**–**F**), or 2-tailed *t* test (**H** and **J**).

In this experiment, we exposed aged mice to transient hypoxia as a tool to enhance macrophage metabolism and inflammatory function immediately following disuse atrophy for 24 hours, thereby improving muscle recovery outcomes. To determine whether acute hypoxic exposure could enhance early functional muscle regrowth in old mice, we first examined metrics of whole-body and muscle-specific force production during the recovery from disuse atrophy (Figure 1A). Body weight recovery patterns were similar across aged mice, with moderate and full return to baseline levels by 7 days of recovery (Figure 1B). Soleus mass was not different from baseline at 4 days of recovery for old hypoxia-treated mice, but all aged mice had similar mass to baseline levels by 7 days (Supplemental Figure 1A; supplemental material available online with this article; https://doi.org/10.1172/jci.insight.194303DS1). Gastrocnemius mass was greater at 4 and 7 days compared with hind limb unloading (HU) but remained lower compared with baseline values for all aged mice (Supplemental Figure 1B). Interestingly, despite substantial loss of grip strength following HU, old mice treated with acute hypoxia at the initiation of reloading experienced rapid strength recovery compared with age-matched controls (Figure 1C). Body weight and gastrocnemius recovery patterns were similar between young control and hypoxia-treated mice (Figure 1D and Supplemental Figure 1, C and D). Interestingly, hypoxia-treated young mice had substantially enhanced grip strength recovery compared with young controls (Figure 1E). While old mice were weaker than young mice at baseline, old hypoxia-treated mice had similar strength to young controls at the 4- and 7-day recovery time points (Supplemental Table 1). Together, these findings demonstrate that acute hypoxia following disuse atrophy improves the recovery of whole-body strength in young and aged mice.

To further explore the enhanced whole-body recovery of strength in old mice, we isolated soleus muscles at 7 days recovery following HU and evaluated ex vivo force production characteristics. Notably, old hypoxia-treated mice displayed greater soleus-specific force generation across a range of frequencies (100–200 Hz) compared with old controls (Figure 1F), with clear separation in representative force tracing at 200 Hz stimulation (Figure 1G). Conversely, maximal twitch force obtained at optimal length was not different between groups (Figure 1, H and I), yet the soleus from old hypoxia-treated mice had greater rates of relaxation compared with age-matched controls (Figure 1J).

### Muscle cellular dynamics are improved in hypoxia-treated mice.

**Figure 2:**
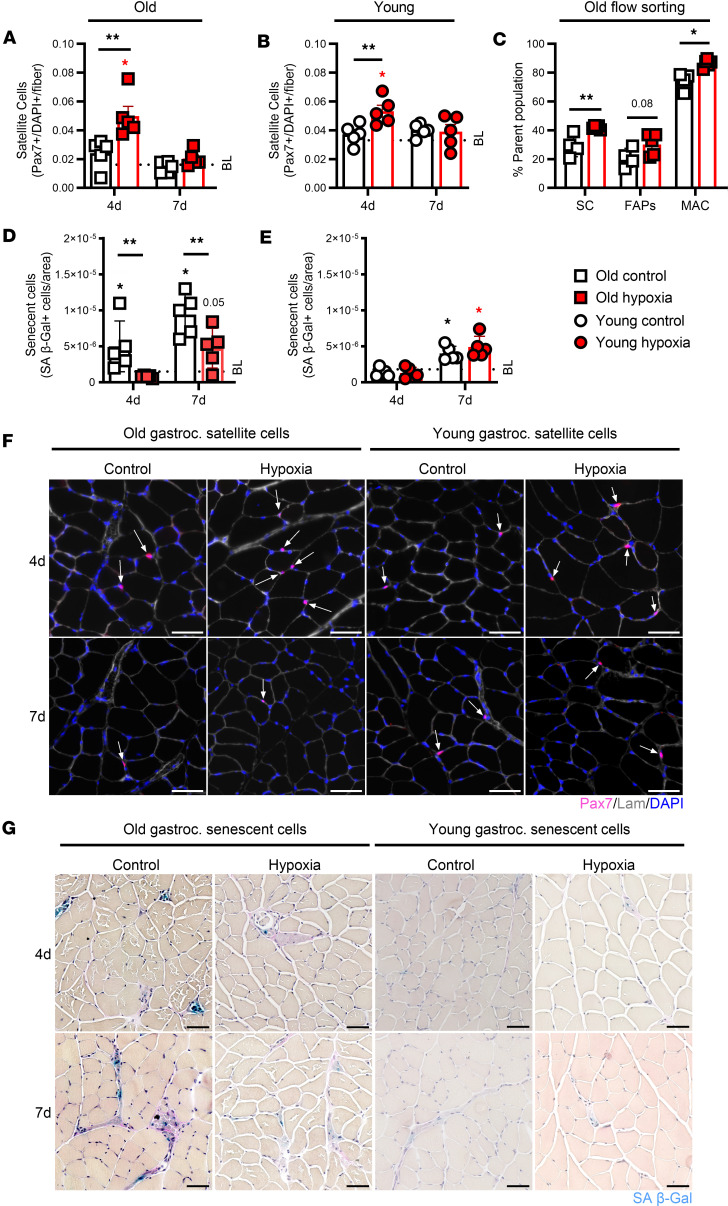
Muscle cellular dynamics. (**A** and **B**) Gastrocnemius satellite cells (Pax7^+^DAPI^+^) per muscle fiber in old and young mice at baseline (BL), and 4 days (4d) and 7d of recovery from hind limb unloading (*n* = 5). (**C**) Gastrocnemius flow cytometry analysis of satellite cells (SCs: CD31^–^CD45^–^α7-integrin^+^), fibro/adipogenic progenitor cells (FAPs: CD31^–^CD45^–^Scal1^+^), and proinflammatory-like macrophages (MAC: CD31^–^CD45^+^F4/80^+^CD206^–^) from old mice at 4d (*n* = 4–5). (**D** and **E**) SA β-Gal^+^ cells per gastrocnemius area in old and young mice (*n* = 5). (**F** and **G**) Satellite (Pax7: pink, DAPI: blue, laminin: gray) and senescent cell (SA β-Gal: blue) representative images at 4d and 7d for old and young mice, with arrows indicating satellite cells. Original magnification, ×20 (**F**) and ×10 (**G**). Scale bars: 50 μm (**F** and **G**). Each data point represents an individual mouse, presented as mean ± SD. *P < 0.05, **P < 0.01 for difference from BL; asterisks over a solid line indicate a difference at those time points. Red color indicates hypoxia exposure, white color indicates control. Significance assessed by 2-way ANOVA with Holm-Bonferroni test. (**A**–**D**) or 2-tailed *t* test (**E**).

To gain greater understanding of the muscular effects of hypoxic treatment during the recovery from disuse, we examined skeletal muscle interstitial cellular dynamics critical for muscle growth and recovery. Old mice treated with hypoxia displayed greater gastrocnemius satellite cell content at 4 days of recovery compared with old controls (Figure 2A). Similarly, young hypoxia-treated (versus young control mice) had higher satellite cell content at 4 days of recovery (Figure 2B). Of note, while satellite cell content was lower in old compared with young mice at baseline, old hypoxia-treated mice had greater satellite cell content compared with young mice at 4 days of recovery (Supplemental Table 2). Flow cytometry of gastrocnemius muscle at 4 days of recovery corroborated the satellite cell immunofluorescence data, demonstrating a greater proportion of satellite cells (α7^+^ cells) compared with old controls and further revealed that old hypoxia-treated mice had a heightened content of proinflammatory-like macrophages (F4/80^+^CD206^–^ cells) (Figure 2C).

We additionally examined the abundance of senescence-associated β-galactosidase–positive (SA β-Gal^+^) cells given that senescent cells accumulate in aging following muscle injury (21). As a result, we found that old hypoxia-treated mice had fewer SA β-Gal^+^ than aged-matched controls at 4 and 7 days of recovery from disuse (Figure 2D). Hypoxia treatment in young mice did not alter the content of SA β-Gal^+^ cells at 7 days of recovery compared to young controls (Figure 2E). Importantly, old hypoxia-treated mice had a similar number of senescent cells during muscle recovery compared with young mice, suggesting a return to youthful levels (Supplemental Table 2). Representative SA B-Gal^+^ satellite cell images are shown in Figure 2, F and G.

In regard to myofiber size, there were no differences in total gastrocnemius myofibrillar CSA (fCSA) at 4 and 7 days of recovery between control and hypoxia-treated aged mice (Supplemental Figure 1, E and F). However, when assessed by fiber type frequency, old hypoxia-treated mice had a moderately greater size at 4 and 7 days for type I and IIb fibers (Supplemental Figure 1, G–L). In young hypoxia-treated mice, there was enhanced recovery of type IIb fibers at 4 days of recovery, while 7-day recovery distributions were similar compared to baseline (Supplemental Figure 1, M–R). In total, these results show that hypoxia treatment following disuse atrophy enhanced muscle cellular dynamics at recovery in old and young mice.

### Acute hypoxia promotes robust collagen remodeling during early muscle regrowth.

**Figure 3:**
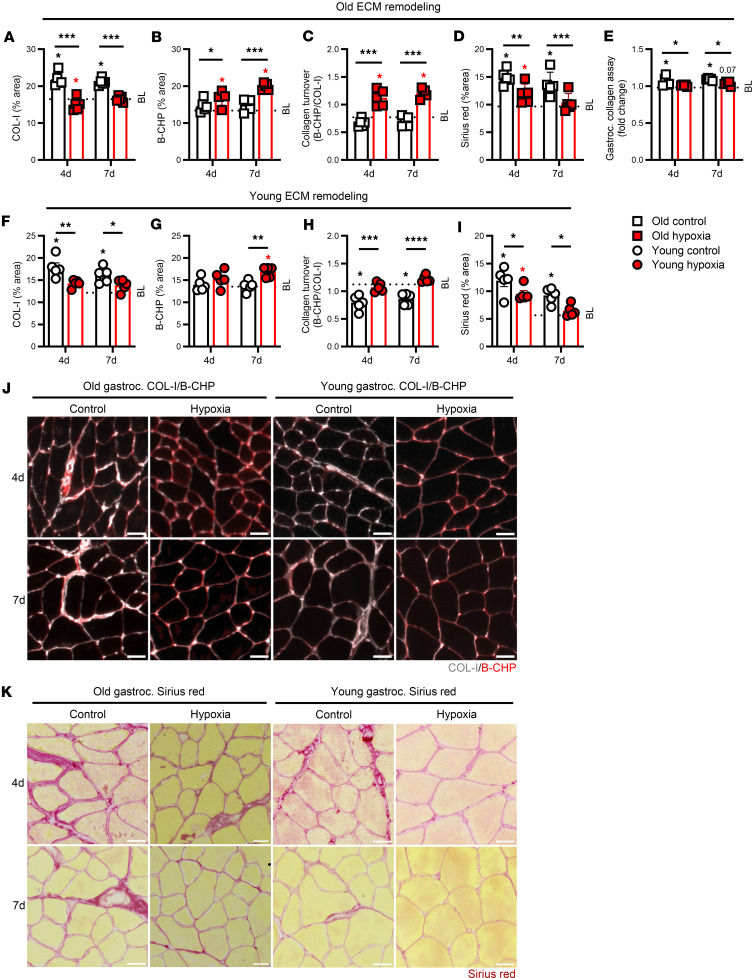
Muscle collagen remodeling. (**A**–**D**) Gastrocnemius type I collagen (COL-I), collagen hybridizing peptide (B-CHP), COL-I/B-CHP ratio, and Sirius red expression at baseline (BL) and at 4 days (4d) and 7d of recovery from hindlimb unloading for old mice (*n* = 5). (**E**) Fold change from BL in total collagen assay content from gastrocnemius lysate at 4d and 7d in old mice (*n* = 5). (**F**–**I**) COL-I, B-CHP, COL-I/B-CHP, and Sirius red expression in young mice (*n* = 5). (**J** and **K**) COL-I/B-CHP (COL-I: red, B-CHP: white) and Sirius red representative images at 4d and 7d for old and young mice. Original magnification, ×20 (**J**) and ×10 (**K**). Scale bars: 25 μm (**J** and **K**). Each data point represents an individual mouse, presented as mean ± SD. Red color indicates hypoxia exposure, white color indicates control. *P < 0.05, **P < 0.01, ***P < 0.001 by 2-way ANOVA with Holm-Bonferroni test for difference from BL; asterisks over a solid line indicate a difference at those time points.

As proinflammatory macrophages and satellite cells influence muscle ECM remodeling (18, 22), we next examined changes in muscle collagen content. At 4 and 7 days of recovery following HU, old hypoxia-treated mice had lower levels of collagen type I (COL-I) expression in gastrocnemius muscle compared with old control mice (Figure 3A). Alternatively, at 4 and 7 days of recovery the gastrocnemius expression of collagen hybridizing peptide (B-CHP; marker of collagen breakdown) in old hypoxia-treated mice was elevated compared with baseline and old control mice (Figure 3B). As a result, we calculated the ratio of collagen turnover (B-CHP/COL-I), which suggested an increased collagen turnover ratio in old hypoxia-treated mice compared with baseline and old control mice (Figure 3C). To further demonstrate that muscle collagen content was impacted by acute hypoxia treatment, we performed Sirius red staining on histological sections and a total collagen/hydroxyproline assay in gastrocnemius lysates. In both approaches, old hypoxia-treated mice had reduced collagen content during the recovery from HU compared with control mice (Figure 3, D and E). Similarly, hypoxia treatment in young mice had lower levels of COL-I at 4 and 7 days of recovery and increased B-CHP expression at 7 days of recovery compared with young controls (Figure 3, F and G). Accordingly, collagen turnover was increased in young hypoxia-treated mice at 4 and 7 days of recovery, while Sirius red content was decreased at 7 days of recovery (Figure 3, H and I). Despite greater baseline collagen content in old versus young mice, old hypoxia-treated mice had similar collagen content (COL-I, Sirius red) at 4 and 7 days of recovery compared with young control mice (Supplemental Table 3). Representative COL-I, B-CHP, and Sirius red images are shown in Figure 3, J and K.

Finally, we conducted correlational analyses to determine whether changes in muscle cellular dynamics and collagen remodeling correspond to each other and functional muscle recovery. We found that at both 4 and 7 days of recovery there were moderate to strong associations between variables, including grip strength, fCSA, satellite and senescent cell content, and collagen levels (Supplemental Figure 2). Therefore, it appears that the hypoxia-stimulated changes to muscle cellular dynamics, collagen content, and functional recovery following acute hypoxic exposure in young and old mice are closely associated.

### Hypoxia directly and indirectly influences cellular function in vitro.

To better elucidate the specific effects whole-body hypoxia had on macrophages, satellite cells, and fibroblasts we utilized murine RAW 264.7 macrophages, C2C12, and NOR-10 cells to help elucidate this scenario in vitro. When stimulated with 1% O_2_ for 24 hours, macrophages had improved phagocytic capacity compared with unstimulated controls (Supplemental Figure 3A). Additionally, macrophages exposed to hypoxia displayed increased expression of HIF1A and IL-6 genes as well as HIF1A but decreased phosphorylated 4EBP1 proteins compared with controls (Supplemental Figure 3, C and D). Hypoxia-treated macrophages were also enriched with relevant proinflammatory metabolites, including succinate, while resolving factors like oleic acid were reduced (Supplemental Figure 3F). Finally, the conditioned media generated from these hypoxia-treated macrophages were also enriched with metabolites that could elicit secondary effects in the local cellular environment (Supplemental Figure 3G).

Conversely, myoblasts and myotubes exposed to hypoxia did not exhibit changes to proliferation or differentiation, respectively, compared to controls (Supplemental Figure 4, A–D). Similarly, few metabolites were differentially enriched in C2C12s treated with hypoxia (Supplemental Figure 4E). To determine whether hypoxia may have secondary effects on satellite cells through macrophages, we treated murine C2C12 cells with the 48-hour culture media from hypoxia-treated and control RAW 264.7 macrophages. Treatment with hypoxic macrophage–conditioned media enhanced the basal proliferation, differentiation, and wound healing capacity compared with control conditioned media (Supplemental Figure 4, A–G). These in vitro experiments agree with previous literature suggesting that severe hypoxia does not directly stimulate proliferation (23) or differentiation of myogenic cells (24). While the proliferation of NOR-10 cells was unaffected by hypoxia treatment, their lipid droplet content was reduced and several metabolites were significantly enriched (Supplemental Figure 4, H–L). Hypoxic macrophage–conditioned media did not influence NOR-10 proliferation but mitigated the accumulation of lipids that control conditioned media appeared to promote (Supplemental Figure 4, H–K). Therefore, our results suggest that macrophages, and to a lesser extent fibroblasts, but not myogenic cells, are functionally and metabolically affected by direct hypoxia in vitro. While we cannot fully recapitulate the effects of hypoxia in vivo (25, 26), our results and others (27) support that macrophages can be targeted with hypoxia and promote secondary effects in muscle cells and fibroblasts.

### The global Raman molecular signature of muscle recovery in aged hypoxia-treated mice recapitulates a young muscle phenotype.

**Figure 4:**
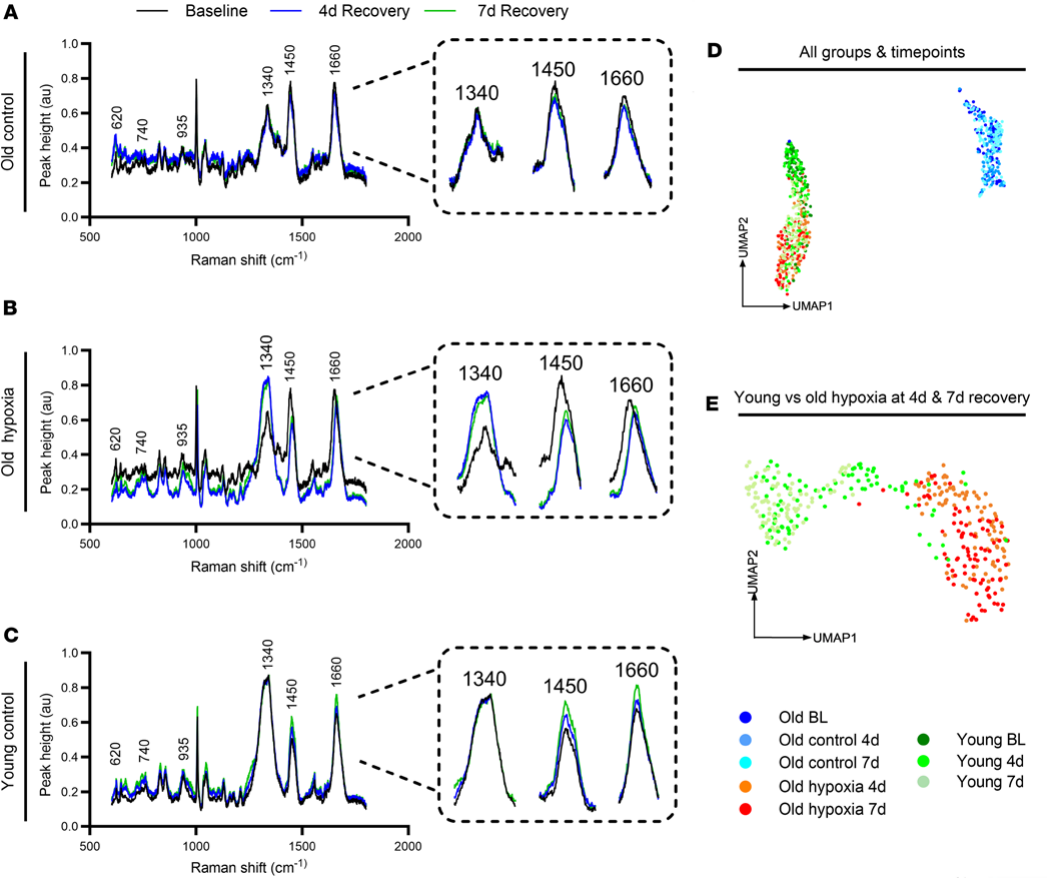
Global Raman molecular signature. (**A**–**C**) Raman spectral signature from 600–1800 cm^–1^ and isolated peaks (AU) at ~1340 cm^–1^, ~1450 cm^–1^, and ~1660 cm^–1^ at baseline (BL), and at 4 days (4d) and 7d of recovery from hind limb unloading for old control, old hypoxia-treated, and young control mice (*n* = 3). (**D**) UMAPs of average Raman spectral distributions at BL, 4d, and 7d of recovery for all mice (**E**) and young and old hypoxia-treated mice at 4d and 7d. Spectral signatures represent average of 30 individual measurements for each sample.

In order to characterize the molecular landscape and biochemical recovery pattern of muscle regrowth, we employed Raman spectroscopy analysis. Raman spectroscopy is a label-free technique that captures the vibrational spectrum of chemical bonds, thus providing a molecular fingerprint that can be used to characterize the biochemical composition of complex biological tissues (28, 29). Furthermore, secondary machine learning analysis (30) of Raman spectral readouts can provide unique insights, considering molecular relationships that have emerging clinical relevance (28, 29). As an example, Raman analysis can provide robust compositional information, considering the molecular signature of various tissue pathologies (31–33). Therefore, we examined the average Raman spectra between 600 and 1800 cm^–1^ and noticed a distinct molecular phenotype that was substantially different between young and aged mice at 4 and 7 days of recovery (Figure 4, A–C). Notably, the molecular signature of old hypoxia-treated mice had remarkable similarity to that of young controls during the recovery from disuse. Specifically, old hypoxia-treated mice displayed distinct changes in Raman regions corresponding to nucleic acids (1340 cm^–1^), lipids (1450 cm^–1^), and proteins (1660 cm^–1^). To further visualize the dimensions of Raman spectral distributions, UMAPs were created to cluster data by group and time. The spectral distributions from old hypoxia-treated mice clustered closely with those of young control mice, while old control mice were quite distinct from either group (Figure 4D). Further examination of the spectra from old hypoxia-treated and young mice at 4 and 7 days of recovery without baseline data demonstrated residual separation between groups despite their overall convergence (Figure 4E). Therefore, we have characterized a Raman molecular phenotype in aged muscle during the recovery from disuse atrophy that can be rejuvenated with hypoxia treatment.

### Raman biochemical relationships correspond to tissue and functional outcomes.

**Figure 5:**
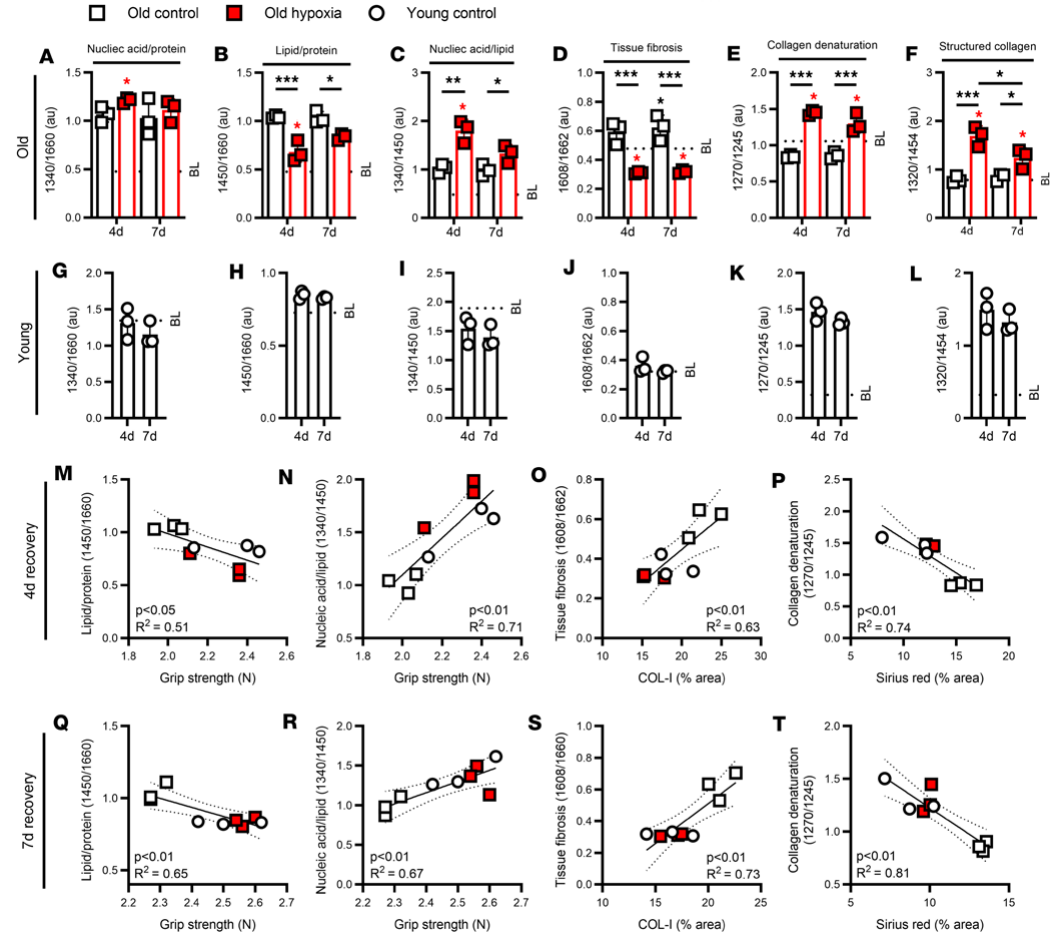
Raman biochemical relationships. (**A**–**F**) Raman spectral ratios for nucleic acid/protein (~1450/1660 cm^–1^), lipid/protein (~1340/1660 cm^–1^), nucleic acid/lipid (~1340/1450 cm^–1^), tissue fibrosis (~1608/1662 cm^–1^), collagen denaturation (~1270/1245 cm^–1^), and structured collagen (~1320/1454 cm^–1^) for old mice at baseline (BL), and at 4 days (4d) and 7d of recovery from hindlimb unloading (*n* = 3). (**G**–**L**) Raman spectral ratios for young mice (*n* = 3). Correlations between lipid/protein ratio and grip strength, nucleic acid/lipid ratio and grip strength, tissue fibrosis ratio and type I collagen, and collagen denaturation ratio and Sirius red for young and old mice at 4d (**M**–**P**) and 7d (**Q**–**T**) of recovery. Spectral signatures derived from average of 30 individual measurements for each sample; each data point represents spectral average or reading from an individual mouse, presented as mean ± SD. Red color indicates hypoxia exposure, white color indicates control. *P < 0.05, **P < 0.01, ***P < 0.001 by 2-way ANOVA with Holm-Bonferroni test for difference from BL (**A**–**L**); asterisks over a line in **A**–**F** indicate a difference between groups at that time point. Significance in **M**–**T** was assessed using Pearson’s correlation.

In order to provide biochemical and physiological relevance to the Raman signatures, we examined the ratio of specific spectra within key regions related to cellular health (31) as well as tissue fibrosis and collagen integrity (32, 33). Interestingly, the muscles of old hypoxia-treated mice had altered ratios of common biochemical constituents, including nucleic acid/protein (~1450/1660 cm^–1^), lipid/protein (~1340/1660 cm^–1^), and nucleic acid/lipid (~1340/1450 cm^–1^) compared with old controls at recovery and baseline time points (Figure 5, A–C). Furthermore, old hypoxia-treated mice had lower fibrosis associated relationships (~1608/1662 cm^–1^) at 4 and 7 days of recovery from disuse compared with old control mice (Figure 5D). Likewise, relationships associated with collagen denaturation (~1270/1245 cm^–1^) and organization (~1320/1454 cm^–1^) displayed altered responses in old hypoxia-treated compared with old baseline and control mice (Figure 5, E and F). Notably, the ratios of old hypoxia-treated mice were similar to those of young mice across each metric (Figure 5, G–L). In agreement, correlation analysis supported that the Raman ratios associated with histological and functional outcomes with clear grouping of young control and old hypoxia-treated mice. At 4 days of recovery, lipid/protein and nucleic acid/lipid ratio were negatively and positively associated with grip strength recovery, respectively (Figure 5, M and N). Additionally, Raman tissue fibrosis was positively associated with type I collagen, and collagen denaturation negatively associated with Sirius red at 4 days of recovery (Figure 5, O and P). Similar directionality and strength of relationships were found at 7 days of recovery (Figure 5, Q–T). These data further corroborate hypoxia-mediated rejuvenation of the aging muscle phenotype during early regrowth and demonstrate strong relationships between biochemical spectral relationships and structural and functional recovery outcomes.

### Proinflammatory metabolic rewiring in aged macrophages from hypoxia-treated mice.

**Figure 6:**
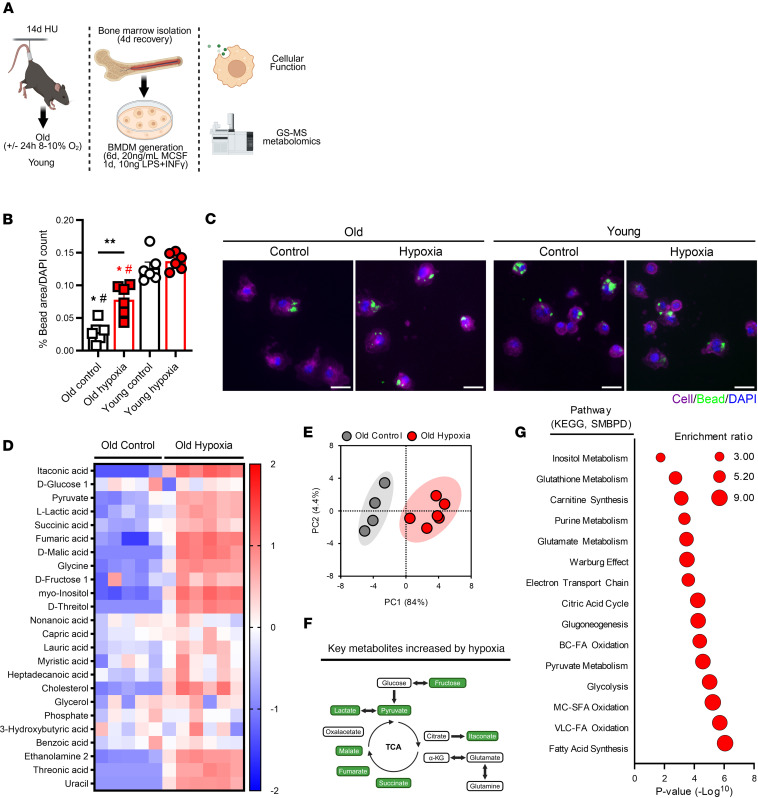
Macrophage function and metabolism. (**A**) Summary of experimental design. (**B** and **C**) Phagocytic capacity of BMDMs from old and young mice isolated at 4 days (4d) of recovery from hind limb unloading (*n* = 6) and representative image (cell: purple, beads: green, DAPI: blue). Original magnification, ×20. Scale bars: 20 μm. Each data point in **B** represents cells from an individual mouse, presented as mean ± SD. **P < 0.01 by 1-way ANOVA with Dunnett’s test for difference from young control; ^#^*P* < 0.05 for difference from young hypoxia. Red color indicates hypoxia exposure, white color indicates control. Asterisks over a line in **B** indicate a difference between groups at that time point. (**D**–**G**) Metabolomics analysis of old BMDMs isolated at 4d of recovery (*n* = 4–6), including metabolite content, PCA distribution, summary of increased glycolytic/TCA-related metabolites, and key enriched metabolic pathways (KEGG and SMBPD). (**D** and **E**) Heatmap and PCA clustering (log_2_[fold change]). (**F**) Significantly increased metabolites (log_2_[fold change] ≥ 1.5, *P*_adj_ ≤ 0.05). (**G**) Significant pathway enrichment (normalized enrichment score ≥ 2, *P* ≤ 1.3 log_2_). *P* < 0.05 or *P* ≤ 1.3 log_2_ was considered significant.

As infiltrating proinflammatory macrophages play a key role in muscle cellular dynamics and remodeling (13, 15), we isolated bone marrow–derived macrophages (BMDMs) from old hypoxia-treated mice and age-matched controls at 4 days of recovery (Figure 6A). Of interest, we found that hypoxia-treated aged mice had a greater capacity for phagocytosis than old controls and that this functional response approached young phagocytic capacity levels (Figure 6, B and C). We then utilized targeted metabolomics to examine changes in metabolite accumulation and pathway responses in these cells. In Figure 6D, we show that BMDMs from hypoxia-treated mice collected at 4 days of recovery had greater levels inflammation-related metabolites and were distinct from old control BMDMs with increased levels of key glycolytic and TCA metabolites, including lactate, itaconate, and succinate (Figure 6, E and F). Additionally, enrichment analysis supported the idea that BMDMs from hypoxia-treated mice had greater stimulation of glycolytic, citric acid cycle, and Warburg effect pathways compared with old controls (Figure 6G).

### The intramuscular delivery of hypoxia-treated macrophages enhances functional muscle recovery in aged mice.

**Figure 7:**
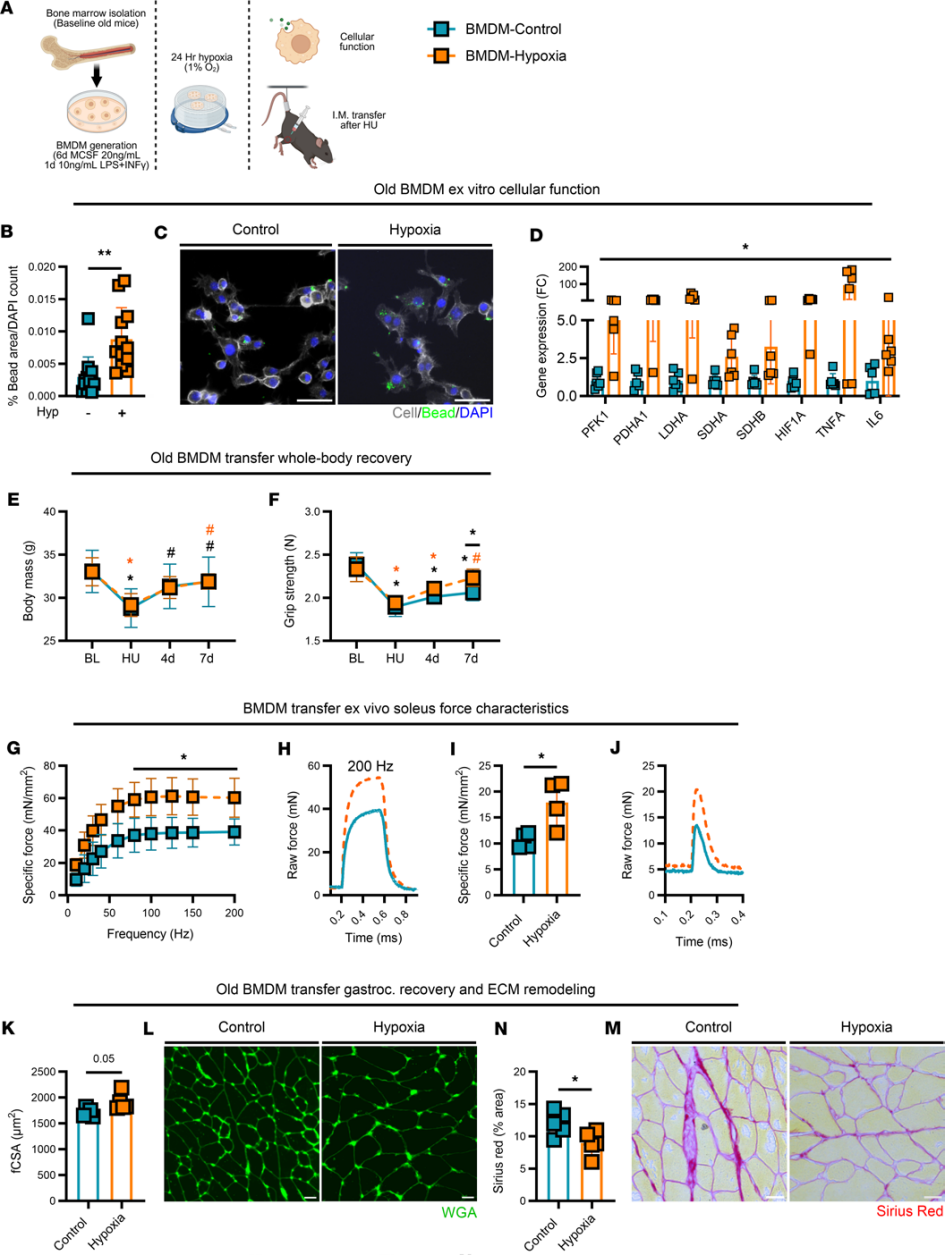
Adoptive transfer of hypoxia-treated macrophages. (**A**) Summary of experimental design. (**B**–**D**) Phagocytic capacity (*n* = 10) and gene expression profile (*n* = 6) of replicate BMDMs isolated from old mice at baseline (BL) with or without ex vitro hypoxia treatment (cell: white, beads: green, DAPI: blue). Original magnification, ×20. Scale bar: 50 μm. (**E** and **F**) Body weight and grip strength (*n* = 5) at BL, following hind limb unloading (HU), and at 4 days (4d) and 7d of recovery from HU in old mice treated with control or hypoxia-stimulated BMDMs. (**G** and **H**) Ex vivo soleus-specific force frequency (Nm/mm^2^) from 10 to 200 Hz (*n* = 4) and representative absolute force tracing (mN) at 200 Hz stimulation. (**I** and **J**) Soleus-specific twitch at optimal length and representative peak twitch tracing. (**K**–**N**) Gastrocnemius fCSA (wheat germ agglutinin [WGA]: green) and Sirius red at 7d of recovery. Original magnification, ×10 (**L**) and ×20 (**N**). Scale bars: 25 μm (**L** and **N**). Each data point represents an individual replicate or mouse, presented as mean ± SD. Orange color indicates hypoxia-treated macrophage injection, teal color indicates control. *P < 0.05 for difference from BL; ^#^*P* < 0.05 for difference from HU. An asterisks over a line in **F** indicates a difference between groups at that time point. Significance was assessed by 2-tailed *t* test (**B**, **D**, **I**, **K**, and **M**) or 2-way ANOVA with Holm-Bonferroni test (**E**–**G**).

To support the notion that macrophages may be directly targeted by whole-body hypoxia in aged mice, we isolated BMDMs from old baseline mouse donors and then exposed these cells to acute in vitro hypoxia (Figure 7A). BMDMs were functionally responsive to hypoxia, as indicated by greater phagocytosis compared with untreated controls (Figure 7, B and C) as well as enhanced glycolytic, TCA, and inflammatory gene expression profiles (Figure 7D). BMDMs were then transferred via intramuscular injection to the triceps surae (gastrocnemius) group to aged control donor mice immediately following the HU period. The intramuscular delivery of hypoxia-stimulated BMDMs immediately following HU had no effect on body mass but improved grip strength recovery in old mice (vs. unstimulated BMDM mice; Figure 7, E and F) such that by 7 days of recovery, mice treated with hypoxia-stimulated BMDMs were not different from baseline. Furthermore, at 7 days of recovery, the specific force of isolated soleus muscles across a range of stimulated frequencies (60–200 Hz) as well as peak twitch at optimal length and their isolated force tracings were greater in aged mice delivered hypoxia-treated BMDMs compared with control nonstimulated-BMDM-treated mice (Figure 7, G–J). Finally, at the whole-muscle histological level at 7 days of recovery, old mice delivered hypoxia-treated BMDMs had greater gastrocnemius fCSA (Figure 7, K and L) and decreased collagen deposition (Figure 7, M and N).

## Discussion

In the current investigation, we demonstrate that acute exposure to hypoxia alters the metabolism of macrophages and stimulates proinflammatory-like macrophage function, cellular remodeling, and early muscle regrowth following disuse atrophy in aged mice. Furthermore, aging muscle has a distinct Raman molecular signature and biochemical recovery pattern during the recovery from disuse atrophy that resembles a younger phenotype following brief exposure to hypoxia. Finally, the intramuscular delivery of hypoxia-treated macrophages recapitulated the beneficial effects of whole-body hypoxia in old mice. These findings emphasize the essential role of proinflammatory macrophages in the aging muscle niche during regrowth from disuse atrophy and highlight that macrophage dysfunction can be reversed through targeted immunometabolism approaches.

Previous work from our lab demonstrated that age-related reductions in glycolytic responsiveness, succinate accumulation, and HIF1A transcription underscore proinflammatory macrophage dysfunction and impaired muscle regrowth in old mice (8). In the current investigation, we demonstrate that transient hypoxic exposure delivered during early regrowth in aged mice promotes critical proinflammatory metabolite accumulation and cellular function in myeloid macrophages, and increases proinflammatory-like macrophage content in skeletal muscle (34, 35). These effects coincide with increased muscle satellite cell content in old hypoxia-treated mice, which is known to be regulated by proinflammatory macrophage–derived chemokines, cytokines (17, 36), and metabolites (37). Similarly, old hypoxia-treated mice had improved muscle collagen remodeling compared with old control mice and similar content to young control mice at 7 days of recovery from disuse despite having greater baseline collagen levels. Proinflammatory macrophages are well recognized for their role in debris clearance and collagen remodeling and may have helped facilitate the ECM remodeling observed in the current investigation (16, 18, 38). These interpretations are supported by our in vitro experiments demonstrating that hypoxia-stimulated macrophages have enhanced proinflammatory transcriptional, metabolic, and functional profiles, while fibroblasts, and particularly muscle primary cells, are less robustly affected. Furthermore, hypoxia-treated macrophages stimulated secondary functional effects in muscle cells, and to a lesser extent fibroblasts, through the secretion of pertinent metabolites following stimulation.

Hypoxic exposure was also able to blunt the accumulation of senescence-associated cells (SA β-Gal^+^) during regrowth in aged muscle, with old hypoxia-treated mice demonstrating similar content to young control mice. Additionally, senescent accumulation was most prevalent in disrupted fibrotic regions that are normally occupied by immune cells such as macrophages and fibroblast-like cells. Macrophages have a dynamic relationship with cellular senescence, acting to clear effected cells yet also contributing to senescent burden in aging and disease (39, 40). For example, inhibition of proinflammatory macrophage polarization impairs the clearance of senescent fibroblasts and increases dermal fibrosis during radiation therapy in mice (40). Similarly, Dungan et al. showed that aged mouse muscle was characterized by increased SA β-Gal^+^ and CD11b^+^ immune cells during regeneration and that elimination of these cells improved regeneration (21). Therefore, though speculative, the reductions in SA β-Gal^+^ cell accumulation in the current study may have been partially mediated by greater macrophage clearance following hypoxia treatment, thereby leading to improved functional recovery following disuse in aging.

Functionally, old untreated mice were characterized by impaired strength recovery, while the whole-body strength of old hypoxia-treated mice was similar to young control mice and their soleus force production enhanced compared with old control mice at 7 days of recovery from disuse. The delivery of aged hypoxia-treated macrophages resulted in remarkably similar improvements in muscle recovery, including enhanced strength, muscle force, and reduced muscle collagen accumulation compared with mice receiving nontreated macrophages. This is in line with our previous work demonstrating that the intramuscular delivery of proinflammatory stimulated young or aged macrophages improved the recovery of muscular force in aged mice (20). Likewise, intramuscular delivery of extracellular vesicles or embryonic stem cell secretome are sufficient to restore muscle recovery outcomes in aged mice (41, 42). Cumulatively, our data suggest that poor metabolic and inflammatory function of myeloid-derived macrophages in aged mice can be reversed with whole-body or cell-specific hypoxia treatment, resulting in greater cellular remodeling and early muscle regrowth, including the restoration of muscle function.

In the current investigation, we also identified distinct Raman molecular phenotypes between young and aged murine skeletal muscle that provide insights into the biochemical process of aging and regrowth. Raman analysis has been used previously to identify pathologic states, including muscular dystrophy (43) and cellular senescence (44), and now muscle aging. Importantly, hypoxia-mediated improvements to proinflammatory macrophage function and muscle regrowth corresponded to a remarkable reversal of this aged molecular phenotype. Moreover, Raman spectral relationships related to biochemical composition (31, 45) and collagen content (32, 33) were correlated with histological and functional outcomes in the same mice. Therefore, it is reasonable to speculate that the enhanced proinflammatory macrophage metabolic and functional effects that corresponded to early cellular remodeling and functional regrowth similarly improved the molecular phenotype recovery observed presently.

Finally, as young mice possess adequate proinflammatory macrophage immunometabolic responsiveness and functionality (8, 12), we were surprised that cellular remodeling and muscle regrowth were also enhanced in young hypoxia-treated mice. These data suggest that there may not be a ceiling for improving proinflammatory immunometabolism in young mice. In agreement, enhancing proinflammatory-like macrophage content improves collagen remodeling and muscle regeneration in young mice (46). Moreover, intramuscular injection of macrophage colony–stimulating factor (MCSF) during recovery from disuse enhanced macrophage muscle content and muscle force production during recovery (47). Conversely, we did not observe improved cellular function in the macrophages of young hypoxia-treated mice during the recovery from disuse. Therefore, the effects of whole-body hypoxia in young mice could be working through different mechanisms than those observed in aged mice. While we did not fully explore this incidental finding, it highlights the potential utility of future immunotherapies to accelerate muscle regrowth in young individuals.

In summary, metabolically stimulating macrophages with transient hypoxia promoted critical cellular and molecular remodeling responses that corresponded to early improvements in functional muscle recovery in aged mice following disuse atrophy. Therefore, the results of the current investigation emphasize the crucial role of macrophages during acute muscle regrowth in aging and further establish immunometabolism as a tool to reverse age-related dysfunction following muscle atrophy.

## Methods

Further information can be found in Supplemental Methods.

### Sex as a biological variable.

Young (4–6 months) and older adult (20–22 months) male mice (C57BL/6, NIA aged colonies) were used for all experiments. Skeletal muscle aging is more robust in aged male mice and may more closely recapitulate the aging phenotype of male and female humans than similarly aged female mice (48). While we cannot demonstrate the relevance of these findings in female mice, there is potentially crossover considering the mechanism of muscle aging, which requires further investigation (49).

### Experimental design.

Mice underwent 14 days of HU followed by 4 or 7 days of ambulatory reloading/recovery or remained as baseline controls (Figure 1A). Immediately following HU, experimental mice were exposed to 24 hours of normobaric hypoxia (8%–10% O_2_) via nitrogen gas displacement while control (no hypoxia) mice returned to standard conditions. At each experimental time point, baseline, HU, 4-day, and 7-day whole-body grip strength was recorded and hind limb muscles isolated for analysis: histology, flow cytometry, and force characteristics. A detailed description of these protocols and analyses is provided in the supplemental material.

### BMDMs and in vitro experiments.

Bone marrow was collected at 4 days of recovery from HU or a pooled baseline aged sample and cultured into BMDMs following 6 days of differentiation (MCSF, 20 ng/mL) and 24 hours stimulation (10 ng/mL lipopolysaccharide and IFN-γ). Hypoxia (in vitro) included standard stimulation or stimulation plus 24 hours of hypoxia (1% O_2_, 5% CO_2_, 94% N_2_). Cultured BMDMs were used to assess phagocytic capacity, GS-MS metabolomics, gene expression, or for intramuscular transfer experiments. Transfer experiments occurred immediately following HU via single unilateral intramuscular injection of untreated or treated BMDMs (2 × 10^6^ cells) to the triceps surae group. Mice were allowed to recover for 7 days, followed by tissue collection and muscle force analysis. Murine C2C12 myoblasts/myotubes, NOR-10 fibroblasts, and RAW 264.7 macrophages were cultured under standard conditions and treated with cellular hypoxia as described above. Following hypoxic exposure, cells were assessed for metabolite content, phagocytic capacity, gene and protein expression, proliferation, differentiation, lipid droplet formation, and migration. C2C12 and NOR-10 cells were additionally treated with RAW 264.7 conditioned media (48-hour generation) for 24 hours to examine the secondary effects.

### Raman spectroscopy.

Spectral measurement was performed on cryosectioned tissues with a Renishaw inViva Raman spectrometer and near-IR (785 mm) microscope at Utah State University as previously reported (45). A total of 30 distinct spectra were collected for each tissue between 600 and 1800 cm^–1^ and the fingerprint averaged for each biological sample. Data processing included baseline correction and normalization (asymmetric least-squares) prior to examination of peak analysis, distribution mapping, ratio quantification, and correlation analysis.

### Statistics.

All data are shown as mean ± SD. Where appropriate, 1-way or 2-way ANOVAs and Pearson correlations were used with Holm-Bonferroni or Dunnett’s multiple-comparison test if warranted. Where missing data points were present or groups were unbalanced, mixed effects analysis was used in place of ANOVA. When comparing 2 groups at a single time point, unpaired and paired *t* tests were used where appropriate. Data sets were assessed visually for normality via Q–Q plots, skewedness, and kurtosis, and tested with Shapiro-Wilks test if necessary. Statistical significance was considered at a *P* value of less than 0.05 unless otherwise stated. GraphPad Prism (v10.3.0) or R/RStudio (v4.4.3/v3.6.0) was utilized for all statistical analyses and figure assembly.

### Study approval.

Mice were housed in the Animal Resources Center at the University of Utah, an NIH-approved and AAALAC-accredited facility. All procedures were approved by the University of Utah Institutional Animal Care and Use Committee.

### Data availability.

Data generated in this study and detailed protocols are available in the Supporting Data Values file and supplemental material. Data and protocols will also be made available upon request.

## Author contributions

ZJF participated in the design and conducting of all experiments, performed data analysis, and prepared the manuscript with MJD. This study was led by MJD, including idea conception, research design, data interpretation, and manuscript preparation. NK, MC, and AZ performed Raman measurements and analysis and assisted in manuscript preparation. KF assisted with muscle force measurement and analysis. PEB performed FACS measurements and RMO assisted with analysis. PEB, EMY, RJC, ASK, and JP assisted with murine and in vitro experiments and participated data interpretation or manuscript editing.

## Funding support

NIH, grant R01AG076075, to MJD and RMO.

NIH/National Institute of Diabetes and Digestive and Kidney Diseases, fellowship 5T32DK091317, to ZJF.

## Supplementary Material

Supplemental data

Unedited blot and gel images

Supporting data values

## Version 1 09/09/2025

In-Press Preview

## Version 2 10/22/2025

Electronic publication
