# Supplementary material for: Macrophage metabolic rewiring rejuvenates muscle Raman signatures and cellular remodeling during regrowth in aged mice
Source: JCI Insight. 2025 Sep 9;10(20):e194303. doi: 10.1172/jci.insight.194303 (PMC12581675; doi:10.1172/jci.insight.194303)
Supplement: Supplemental data [file jciinsight-10-194303-s240.pdf]

Supplemental material

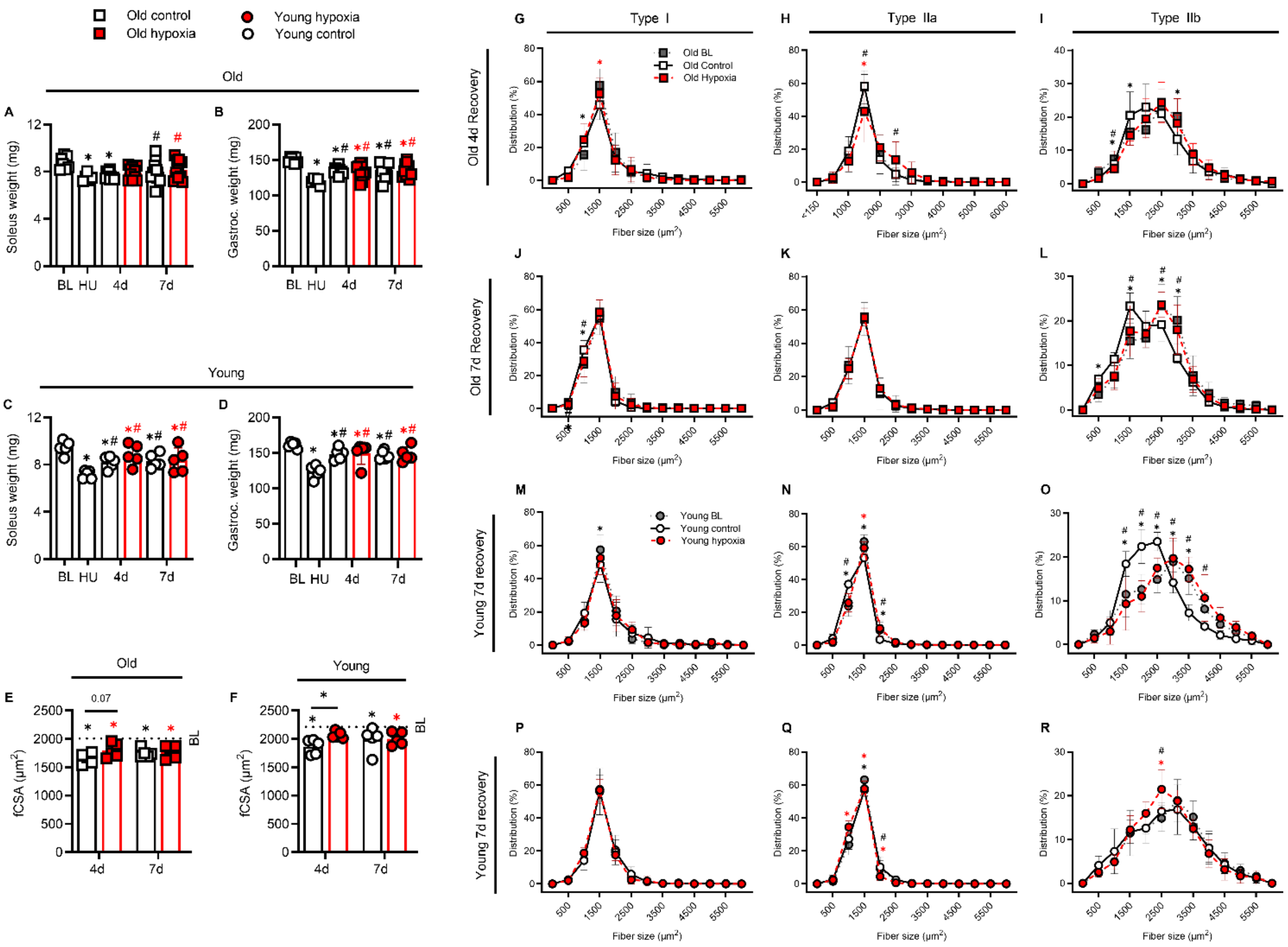

Supplemental Figure 1. Muscle mass and fiber type distribution. (**A-D**) Soleus and gastrocnemius muscle mass at baseline (BL), following hindlimb unloading (HU), and 4d & 7d recovery from HU for old and young mice (n=5-10). (**E** and F**)** Myofibrillar CSA (fCSA) at BL and 4d & 7d recovery from HU for old and young mice (n=5). (**G-R**) Gastrocnemius fiber type specific (type I, IIa, IIb) size distribution at BL and 4d and 7d recovery for old and young mice (n=5). (A-F) Each data point represents an individual mouse, (G-R) data point represents group average, presented as mean ± SD. Two-way ANOVAs, (A-F) *: difference from BL, #: difference from HU, * over line: difference between groups at indicated point. (G-R) *: difference from BL, #: difference between control and hypoxia-treated groups. */# $p<0.05$. $p<0.05$ was considered significant.

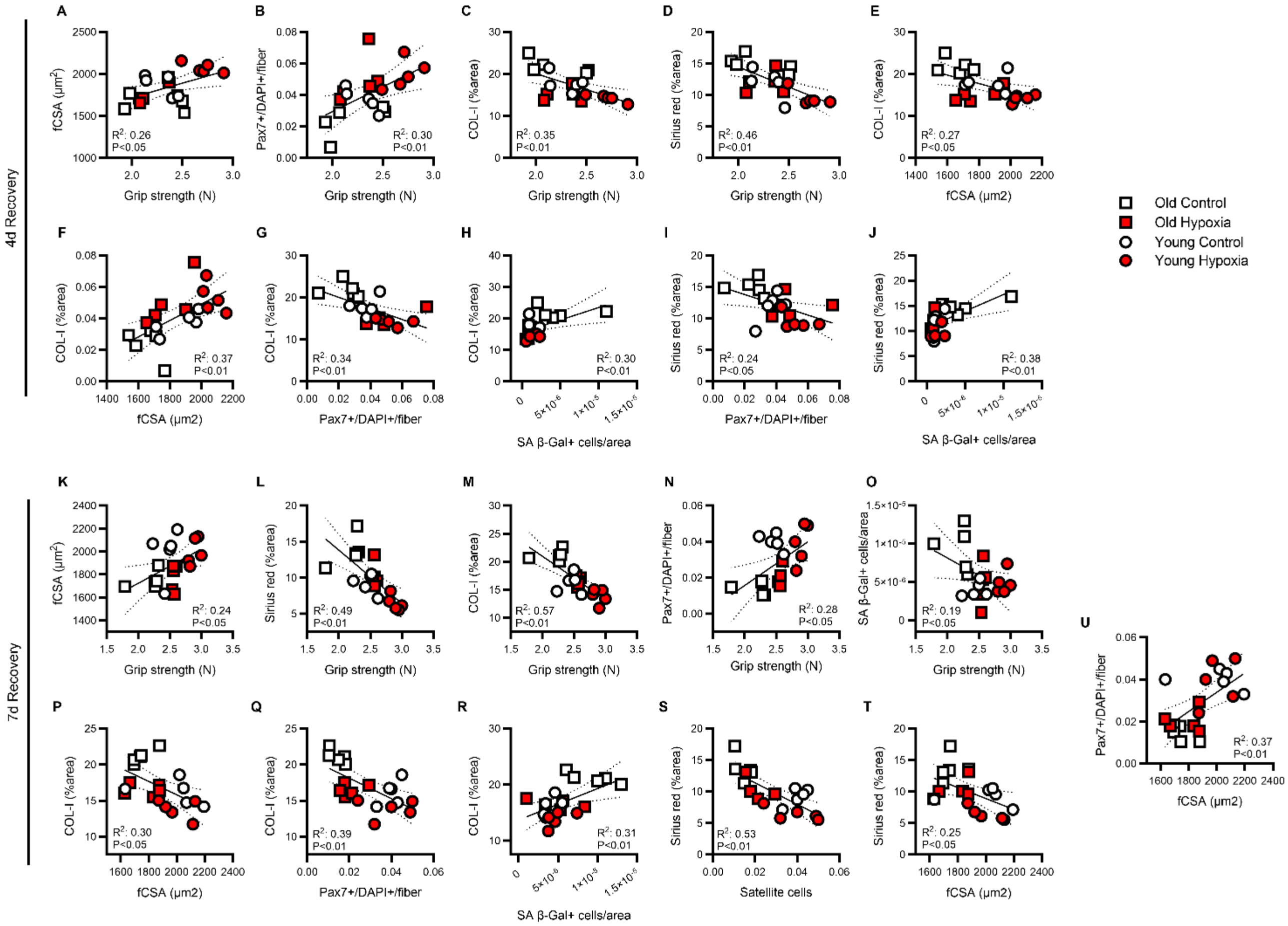

Supplemental Figure 2. Muscle cellular and functional correlations. (**A-U**) Correlation analysis between immunohistochemical, histological, and function outcomes; myofiber CSA (fCSA), satellite cells (Pax7+/DAPI+/fiber), collagen type I (COL-I), Sirius red, senescent cells (SA β-Gal+ cells/fiber) and grip strength (N); measures across the same old and young mice with and without acute hypoxia-treatment immediately following disuse atrophy (n:5). Each data point represents an individual mouse. Analyzed with Pearson's correlations, p-value and R^2 for relationships displayed within each graph. $p<0.05$ was considered significant.

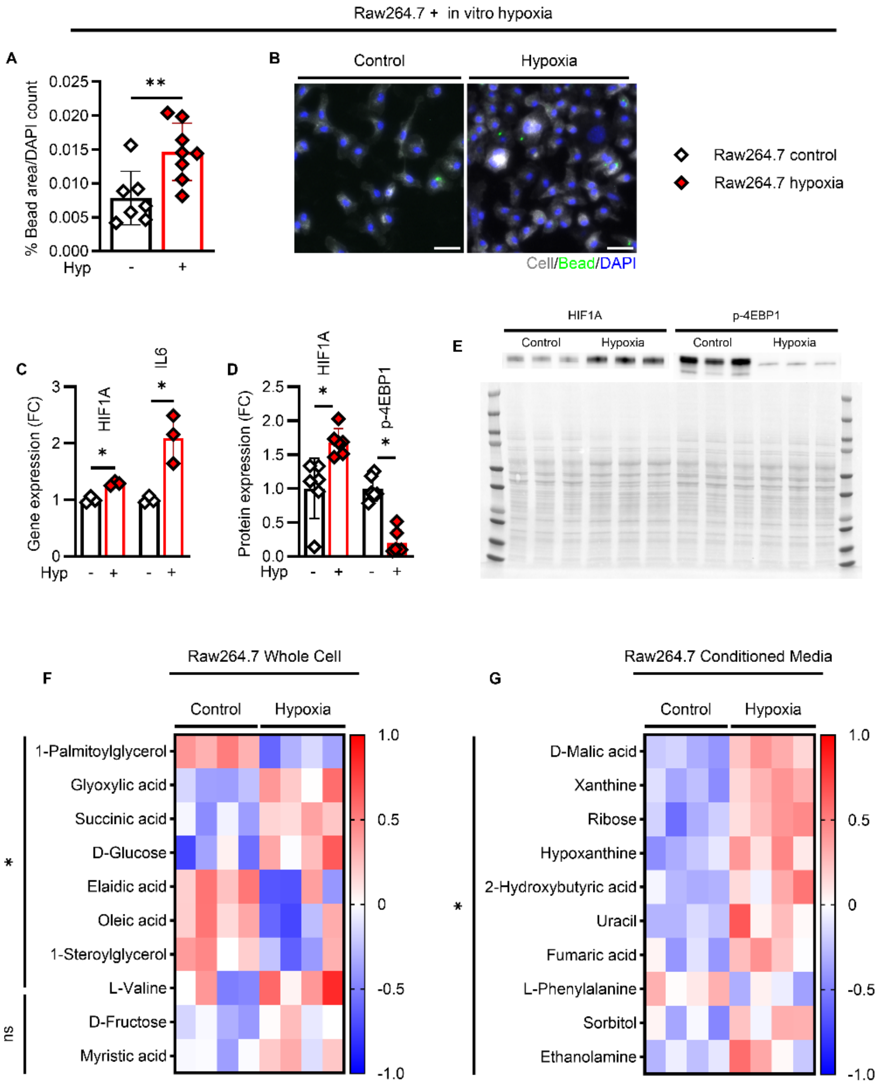

Supplemental Figure 3. In vitro macrophage experiments. (**A and B**) Phagocytosis capacity in Raw264.7 cells (cell: white, beads: green, DAPI: blue, 20X, scale: 20 μm) under standard conditions or treated with acute hypoxia (1% O_2, 5% CO_2, 94% N, 24hr). (**C-E**) gene and protein expression in Raw264.7 cells in control or hypoxia conditions. (**F** and **G**) Metabolite content of hypoxia-treated Raw264.7 cells and their 48-hour post-treatment conditioned media. (A-D) Each data point represents an individual replicate, presented as mean ± SD, two-tailed T-tests, * over line indicates significant difference between groups at indicated point, * $p<0.05$, ** $p<0.01$. (F and G) Altered metabolites, * over line indicates significant difference between groups (≥1.5 log2 fold change, ≤0.05 p-adj.). $p<0.05$ was considered significant.

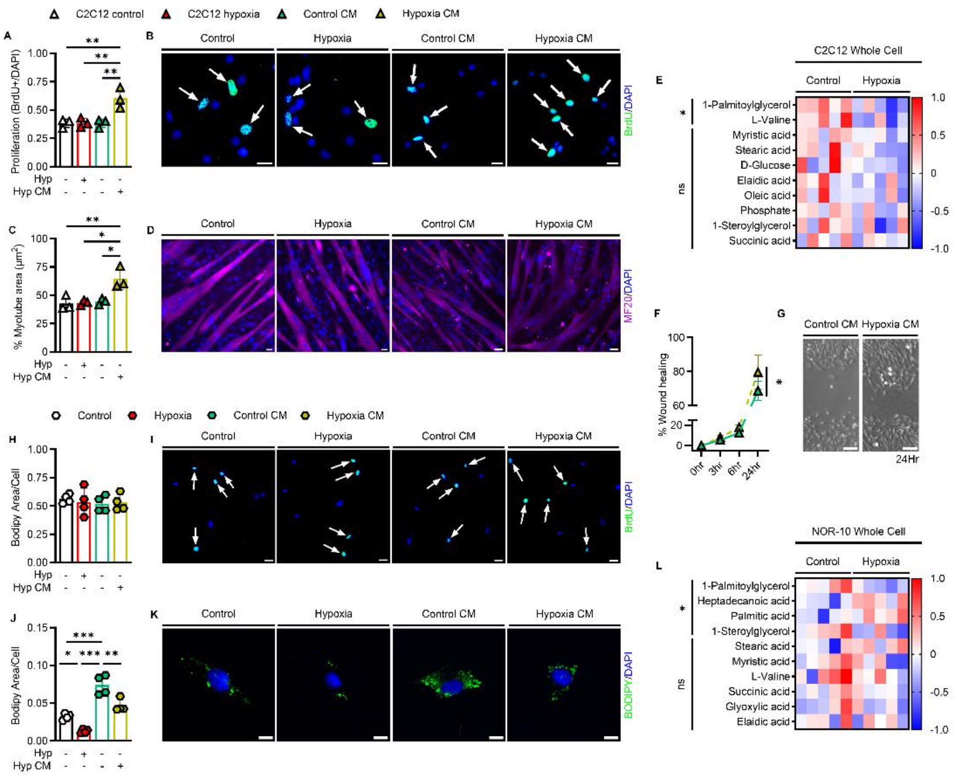

Supplemental Figure 4. In vitro muscle and fibroblast experiments. (**A-D**) Cellular proliferation (BrdU: green, DAPI: blue, 20X, scale: 20 μm) and differentiation (myotubes: purple, DAPI: blue, 20X scale: 20 μm) in C2C12 myoblasts under standard conditions, treated with acute hypoxia (1% O_2, 5% CO_2, 94% N, 24hr), and the 48-hour conditioned media (CM) from untreated or hypoxia-treated Raw264.7 macrophages. (**E**) Metabolite content of hypoxia-treated C2C12 cells. (**F** and **G**) Migrating/wound healing in C2C12 myoblasts treated with the CM from untreated and hypoxia-treated macrophages (Scale: 50 μm). (**H-K**) Cellular proliferation and lipid droplet formation (DAPI: blue, BODIPY: green, scale: 50 μm) in NOR-10 fibroblasts under standard conditions, treated with acute hypoxia, and the 48-hour CM from untreated or hypoxia-treated macrophages. (**L**) Metabolite content of hypoxia-treated NOR-10 cells. (A-D and F-K) Each data point represents an individual replicate, presented as mean ± SD, One-way ANOVA or two-tailed T-tests, * over line indicates significant difference between groups at indicated point, * $p<0.05$, ** $p<0.01$. (E and L) altered metabolites, * over line indicates significant difference between groups (≥1.5 log2 fold change, ≤0.05 p-adj.). $p<0.05$ was considered significant.

Supplemental table 1. Tissue weights & grip strength

	(n=6-20)	Old Control	Old Hypoxia	Young Control	Young Hypoxia
Body Weight (g)	Baseline	33.8 ± 3.3*#	33.5 ± 2.0*#	30.8 ± 1.7	30.5 ± 2.4
	Hindlimb Unloading	30.2 ± 2.0*#	30.0 ± 2.5*#	27.4 ± 1.5	27.8 ± 2.0
	4-Days Recovery	31.8 ± 3.2*#	31.4 ± 2.5*#	29.3 ± 1.3	29.1 ± 2.3
	7-Days Recovery	32.4 ± 1.9*#	32.7 ± 1.9*#	28.9 ± 1.5	28.3 ± 2.0
	(n=6-20)				
Grip Strength (N)	Baseline	2.55 ± 0.22*#	2.49 ± 0.22*#	2.90 ± 0.22	2.93 ± 0.23
	Hindlimb Unloading	2.20 ± 0.21	2.18 ± 0.24	2.30 ± 0.18	2.33 ± 0.15
	4-Days Recovery	2.19 ± 0.20*#	2.31 ± 0.25#	2.41 ± 0.17	2.75 ± 0.26
	7-Days Recovery	2.15 ± 0.19*#	2.46 ± 0.27#	2.47 ± 0.14	2.90 ± 0.08
	(n=5-10)				
Gastrocnemius Weight (mg)	Baseline	150.3 ± 4.3*		161.2 ± 4.3	
	Hindlimb Unloading	120.1 ± 4.5		123.7 ± 9.1	
	4-Days Recovery	134.4 ± 4.8#	133.9 ± 10.8#	148.5 ± 8.5	149.5 ± 15.7
	7-Days Recovery	135.4 ± 11.3	135.6 ± 9.6	146.9 ± 6.3	147.8 ± 10.6
	(n=5-10)				
Soleus Weight (mg)	Baseline	8.7 ± 0.5		9.4 ± 0.6	
	Hindlimb Unloading	7.5 ± 0.3		7.2 ± 0.3	
	4-Days Recovery	7.6 ± 0.3#	7.8 ± 0.5#	8.2 ± 0.5	8.2 ± 0.9
	7-Days Recovery	8.0 ± 0.9	8.1 ± 0.8	8.3 ± 0.6	8.4 ± 1.1

Results from old and young control and hypoxia treated mice at baseline, following 14-days of hindlimb unloading, and 4- and 7-days of recovery from hindlimb unloading. Body weight: grams, gastrocnemius and soleus mass: milligrams, whole-body grip strength: newtons. All values reported as mean ± SD. *: significant difference from young control mice, #: significant difference from young hypoxia mice at respective timepoint. $p<0.05$ was considered significant.

Supplemental table 2. Muscle histology

	(n=5)	Old Control	Old Hypoxia	Young Control	Young Hypoxia
Myofibrillar CSA (μm^2)	Baseline	2006.2 ± 125.4*		2211.0 ± 124.5	
	4-Days Recovery	1656.7 ± 93.7*#	1793.0 ± 129.6#	1863.9 ± 130.1	2070.2 ± 60.8
	7-Days Recovery	1750.0 ± 75.4*#	1776.14 ± 119.0*#	1992.4 ± 211.8	2001.3 ± 116.5
	(n=5)				
Satellite Cells ($Pax7^+/DAPI^+$/Area)	Baseline	0.016 ± 0.007*		0.034 ± 0.003	
	4-Days Recovery	0.024 ± 0.014*#	0.050 ± 0.015*	0.037 ± 0.007	0.053 ± 0.009
	7-Days Recovery	0.014 ± 0.004*#	0.020 ± 0.005*#	0.040 ± 0.005	0.039 ± 0.011
	(n=5)				
Senescent Cells (SA β-Gal+/Area)	Baseline	$1.54 \times 10^{-6} \pm 4.45 \times 10^{-7}$		$1.82 \times 10^{-6} \pm 6.98 \times 10^{-7}$	
	4-Days Recovery	$5.00 \times 10^{-6} \pm 0.35 \times 10^{-7}$ *#	$0.80 \times 10^{-6} \pm 1.87 \times 10^{-7}$	$1.34 \times 10^{-6} \pm 5.90 \times 10^{-7}$	$1.30 \times 10^{-6} \pm 7.65 \times 10^{-7}$
	7-Days Recovery	$9.40 \times 10^{-6} \pm 0.28 \times 10^{-7}$ *#	$4.74 \times 10^{-6} \pm 0.27 \times 10^{-7}$	$4.02 \times 10^{-6} \pm 9.99 \times 10^{-7}$	$4.90 \times 10^{-6} \pm 1.49 \times 10^{-6}$

Results from old and young control and hypoxia treated mice at baseline, following 14-days of hindlimb unloading, and 4- and 7-days of recovery from hindlimb unloading. Myofibrillar cross-sectional area (CSA): μm^2, satellite cells: number of $Pax7^+/DAPI^+$ cells corrected to total myofiber count, senescent cells: Beta-galactosidase (SA β-Gal) positive cells corrected to total muscle area. All values reported as mean ± standard deviation. *: significant difference from young-control mice, #: significant difference from young-hypoxia treated mice, at respective timepoint. $p<0.05$ was considered significant.

Supplemental table 3. Muscle collagen

	(n=5)	Old Control	Old Hypoxia	Young Control	Young Hypoxia
COL-I (%Area)	Baseline	17.3 ± 0.6*		12.1 ± 1.2	
	4-Days Recovery	21.8 ± 1.8*#	15.0 ± 1.7	17.8 ± 2.2	14.2 ± 0.86
	7-Days Recovery	21.1 ± 0.9*#	16.5 ± 0.8#	15.9 ± 1.7	13.8 ± 1.3
	(n=5)				
B-CHP (%Area)	Baseline	13.3 ± 1.6		13.5 ± 0.7	
	4-Days Recovery	14.6 ± 1.6	16.8 ± 1.9*	13.9 ± 1.4	15.3 ± 1.9
	7-Days Recovery	14.9 ± 1.5	19.9 ± 0.9*#	13.6 ± 1.2	16.9 ± 1.2
	(n=5)				
COL-I/B-CHP (au)	Baseline	1.24 ± 0.10*		0.92 ± 0.13	
	4-Days Recovery	1.50 ± 0.14#	0.90 ± 0.11*	1.31 ± 0.26	0.95 ± 0.08
	7-Days Recovery	1.40 ± 0.22#	0.83 ± 0.06*	1.19 ± 0.17	0.83 ± 0.03
	(n=5)				
Sirius red (%Area)	Baseline	9.6 ± 0.2*		5.6 ± 1.0	
	4-Days Recovery	14.9 ± 1.3*#	11.9 ± 1.7	11.9 ± 2.3	9.5 ± 1.3
	7-Days Recovery	13.7 ± 2.1*#	10.3 ± 1.6#	9.2 ± 1.3	6.5 ± 1.0

Results from old and young control and hypoxia treated mice at baseline, following 14-days of hindlimb unloading, and 4- and 7-days of recovery from hindlimb unloading. Collagen type-I (COL-I): % muscle area, collagen hybridizing peptide (B-CHP): % muscle area, COL-I/B-CHP = arbitrary ratio units, Sirius red: % muscle area. All values reported as mean ± standard deviation. *: significant difference from young-control mice at respective timepoint. $p<0.05$ was considered significant.

Supplemental methods

Animals and experimental design. Mice (C57BL/6) were obtained from the NIA aged rodent colonies including young adult (4-6 months) and older adults (20-22 months). Mice had ad libitum access to food and water, were housed (2-4 animals/cage) on a 12/12 hours light/dark cycle, and acclimated ≥1 week prior to experiments. Mice were grouped in a balanced fashion and randomly assigned to groups for all experiments. All procedures complied with the standards of the Institutional Animal Care and Use Committee at the University of Utah. Baseline (BL) mice remained freely ambulatory and received no treatments. To induce disuse atrophy mice underwent 14 days of hindlimb unloading (HU) followed by 4 (4d) or 7 (7d) days of ambulatory reloading/recovery (**Figure 1A**). These have been previously indicated as key time points during the regrowth process (1-3). HU was performed as previously reported (4) using a modified Morey-Holton tail suspension design with two mice per cage. Mouse appearance, weight, food, and water consumption were closely monitored during HU. Immediately following HU, experimental mice were exposed to 24 hours of normobaric hypoxia (8-10% O_2) via nitrogen gas (100%) displacement in a custom sealed and flow regulated chamber equipped with a gas sensor (ProOx110, Biospherix, Parish, NY, USA) while control (no hypoxia) mice returned to standard conditions. Following HU and/or treatment, mice were allowed to freely recover until experimental endpoints.

Tissue collection and analysis. Throughout the experiment body weight and whole-body grip strength (average of top three pulls) were recorded across groups (Columbus Instruments, Columbus OH, USA). At each experimental timepoint; BL, HU, 4d and 7d; hindlimb muscles (gastrocnemius and soleus) were isolated for various analyses. Young (n=5 per group) and old (n=10) gastrocnemius muscles were collected following euthanasia (cervical dislocation) at each timepoint for weight and histological analyses (cross-sectional area, fiber type, satellite cells, collagen remodeling, senescence). A subset of old (n=5-6) mice had both gastrocnemius muscles isolated at 4d for flow cytometry analysis (satellite cells, FAPs, macrophages). An additional subset of old (n=4-6) mice had their soleus muscle

isolated at 7d under local anesthesia (isoflurane) to examine muscle specific force characteristics as previously reported (4).

Muscle force analysis. Suture string was secured to soleus tendons and tied into a horizontal bath tissue system (Model 400A Aurora Scientific Inc., Ontario, CA, USA) equipped with Aurora Scientific Dynamic Muscle Control software (v5.5) filled with Krebs-Ringer solution (8mM glucose) at 37°C supplemented with oxygen. Following 5 min equilibration, optimal muscle length was determined using single pulses (20V, 0.2 ms) with 30 sec rest between stimulations. Following buffer replacement force frequency analysis was performed across 10-200Hz (20V, 0.2ms) with 2 min rest between stimulations. Data analysis was performed using Aurora Scientific Dynamic Muscle Analysis software (v.5.321). To determine specific force (mN/mm^2), cross-sectional area was estimated utilizing muscle weight, length, as well as assumed density (1.06 g/cm^3) (5) and myofiber/muscle length ratio (0.69) (6). Average rates of contraction and relaxation were calculated between 20-80% of peak force.

Immunohistochemistry and histology. Frozen OCT-embedded gastrocnemius muscles were cut in a longitudinal plane at 10µm using a Leica cryostat at -20ºC (CM1860, Leica, Wetzlar, Hesse, Germany). Sections were air dried for 1 hour then maintained at -80ºC prior to staining. Sections were imaged using a Zeiss Slide Scanner Axio Scan.Z1 (Carl Zeiss Inc., Oberkochen, DE) at 10-20x using fluorescent or brightfield capabilities. Images were adjusted for contrast and brightness using Fiji software (v2.9.0) and analyzed as previously described (4, 7) and by using semi-automated techniques via the muscleJ 2.0 plugin as described in detail elsewhere (8).

Analysis	Protocol Summary
fCSA & fiber type	• Blocking: 1 hour Mouse on Mouse (M.O.M.) (MKB-2212-1, Vector Laboratories, Newark, CA, USA) in PBS.<br>• Laminin 1:200 overnight 4ºC (L9393, Sigma-Aldrich, St. Louise, MO, USA).

	• MyHC I 1:100 overnight 4ºC (BA-D5, DSHB, Iowa City, IA, USA).<br>• MyHC IIa 1:100 overnight 4ºC (SC-71, DSHB).<br>• MyHC IIb 1:100 overnight 4ºC (BF-F3, DSHB).
Satellite cells	• Fixing: 10 min PFA (4%).<br>• Antigen retrieval: sodium citrate buffer from 65-92°C for 20 min, cooled to RT.<br>Blocking: 10 min H_2O_2 (3%) followed by 1h in M.O.M. in 1% HS in PBS.<br>• Pax7 1:100 (Pax7c, DSHB) overnight 4ºC.<br>• Tyramide SuperBoost Kit Alexa Fluor 568 (B40956, Invitrogen, Waltham, MA, USA) to amplify the signal.<br>• Laminin 1:200 overnight 4ºC.
Collagen type I (COL-I) and collagen hybridizing peptide (B-CHP)	• Fixing: 10 min acetone at -20ºC.<br>• Blocking: 1 hour 1% HS in PBS.<br>• B-CHP 15 µM overnight (3-Helix, Salt lake City, UT, USA) pre-incubated at 80 ºC for 5 min then cooled prior.<br>• COL-I 1:100 overnight (Abcam, Cambridge, UK).
Sirius Red (collagen types I & III)	• Fixing: 1 hour in Bouin's solution at 56ºC.<br>• Picro Sirius Red 1 hour (ab246832, Abcam).<br>• 3x Acetic acid washes (0.5%), 20s ethanol dehydration (90%, 95%, 100%), Xylene rinse, mounting with Cytoseal XYL (83124, Thermo Fisher, Waltham, MA, USA).
Senescent cells (Adapted from Dungan et al. 2020 (9))	• Fixing: 5min Glutaraldehyde (0.5%).<br>• Staining Solution 72h, changed every 24h: X-gal (1 mg/mL) in DMF, 5mM potassium ferrocyanide, 5mM potassium ferricyanide, 5M NaCl, 1M MgCl2 0.2M cirtic acid/Na phosphate buffer (pH 6.0).

	• Up to 24h PBS washing to remove Na-crystals.<br>• Post-Fixing: 10 min Glutaraldehyde (0.5%).<br>• H&E staining.

Real-Time PCR. RNA was isolated using Qiazol reagent (79306, Qiagen, Hilden, DE) with chloroform and isopropanol extraction per manufacturer recommendations and resuspended in nuclease free water. 1 µg of RNA (EPOCH, TAKE3 BioTek) was used to reverse transcribe 20 µl cDNA (iScript cDNA, Bio-Rad, Hercules, CA, USA) in a thermocycler using the following protocol: 25°C 5 min, 46°C 20 min, 95°C 1 min, 4°C (T100, Bio-Rad). Real-time PCR was performed with cDNA (1:8 in nuclease-free water), 2.5 µM forward and reverse primers, and SsoAdvanced Universal SYBR Green Supermix (Bio-Rad) in a PCR system (CXF Connect, Bio-Rad) per manufacturers recommendations. All data were normalized to ribosomal protein L32 gene expression, quantified using the delta-delta Ct ($2^{-\Delta\Delta Ct}$) method considering fold change from relevant controls, and presented as calculated or in $\log^{10}$ as stated. Gene primers were generated at the University of Utah DNA/Peptide Synthesis Core and are listed in the table below.

Gene ID	Forward (5'-3')/Reverse (3'-5')
L32	TTCCTGGTCCACAATGTCAA/<br>GGCTTTTCGGTTCTTAGAGGA
PFK1	GGAGGCGAGAACATCAAGCC/<br>CGGCCTTCCCTCGTAGTGA
PDHA1	GAAATGTGACCTTCATCGGCT/<br>TGATCCGCCTTTAGCTCCATC
LDHA	TGTCTCAGCAAAGACTACTGT/<br>GACTGTACTTGACAATGTTGGGA

SDHA	GAGATACGCACCTGTTGCCAAG/ GGTAGACGTGATCTTTCTCAGGG
SDHB	TGCGGACCTATGGTGTTGGATG/ CCAGAGTATTGCCTCCGTTGATG
HIF1A	GCTTACACACAGAAATGGCC/ CCTTCCACGTTGCTGACTTG
TNFA	GGCAGGTCTACTTTGGAGTCATTGC/ ACATTCGAGGCTCCAGTGAATTCGG
IL6	TCCTCTCTGCAAGAGACTTCC/ GGAGAGCATTGGAAATTGGGG

Immunoblotting. Proteins were isolated in ice-cold lysis buffer [50 mM Tris-HCl pH 7.5, 150 mM NaCl, 5 mM EDTA, 1% Triton X-100, 0.1% sodium deoxycholate, 0.1% SDS, 1X protease and phosphatase inhibitor (Halt, ThermoFisher, Waltam, MA, USA)]. Supernatants were collected following centrifugation at 12,000 g for 15 min at 4°C. Protein concentrations were determined via assay per manufacturer recommendations (Pierce BCA, ThermoScientific), loaded equally (20ug) and separated by electrophoresis using polyacrylamide gels (4-20%), then transferred to nitrocellulose membranes (Bio-Rad). Ponceau S (K793, VWR, Randor, PA, USA) staining was used to visually confirm protein transfer efficiency. Membranes were blocked for 1 hr in 5% BSA-TSBT at room temperature followed by 3x washes and overnight incubation at 4°C with primary antibodies in 5% BSA-TBST. Following 3x washes, membranes were incubated with secondary antibodies (Cell Signaling anti-mouse 7076 and anti-rabbit 7074, CST, Danvers, MA, USA) at 1:2000 in 5% BSA-TBST for 1 hr at room temperature. Membranes were briefly incubated with ECL Prime Western Blotting Detection Reagent (RPN2236, GE Healthcare, Chicago, IL, USA) then imaged using a ChemiDoc Imaging System (Bio-Rad) and quantified with Image Lab Software (Bio-Rad). Individual proteins were corrected to background and loading controls (GAPDH or Ponceau S). All values were expressed as fold change compared to

relevant control groups. The following primary antibodies were utilized: HIF-1α (C-Term) at 1:500 (10006421, Cayman Chemical, Ann Arbor, MI, USA), phospho-4E-BP1 (Thr37/46) at 1:1000 (2855, Cell Signaling Technology, Danvers, MA, USA).

Raman spectroscopy. Spectral measurement was performed on cryosectioned tissues with a Renishaw inViva Raman spectrometer and near-IR (785 mm) microscope as previously reported (10). A total of 30 distinct spectra were collected for each tissue between 600-1800 cm^{-1} and the fingerprint averaged for each biological sample. Data processing included baseline correction and normalization (asymmetric least-squares) prior to examination of peak analysis, distribution mapping, ratio quantification, and correlation analysis. Tissue fibrosis was assessed by examining the ratio of type I collagen spectra (~1608 cm^{-1}) to the highest peak from the amide I band region (~1662 cm^{-1}) (11), collagen denaturation as the ratio of unordered/random collagen (~1320 cm^{-1}) to the highest peak from the amide III band region (~1454 cm^{-1}), and collagen structural organization as the ratio of unordered/random (~1270 cm^{-1}) to ordered collagen structures (~1245 cm^{-1}) (12). Protein confirmational relationships were assessed by examining the ratio of α-helix (~1652 cm^{-1}), β-sheet (~1663 cm^{-1}), and (~1677 cm^{-1}) nonregular associated protein structural spectra (13).

Bone marrow-derived macrophage experiments. Bone marrow was collected post-euthanasia from the hindlimb bones (femur and tibia) of mice as previously described (3) and cultured into bone marrow-derived macrophages (BMDMs) following 6 days differentiation (MCSF, 20ng/mL) and 24 hours stimulation (10ng/mL lipopolysaccharide and interferon-γ). At 4d recovery BMDMs from young (n=6 per group) and old (n=5-6) mice were assessed for phagocytosis capacity (Cayman Chemical) while old BMDMs were additionally collected (≥$3x10^6$ cells) for targeted gas chromatography-mass spectrometry metabolomics at the University of Utah Metabolomics Core. A subset of BMDM's were collected from old BL mice (n=3) and pooled to create a representative sample using the methods previously described. Following differentiation BMDM's either received standard stimulation or stimulation plus 24 hours nomrobaric hypoxia (1% O_2, 5% CO_2, 94% N) using a hypoxia incubator chamber (STEMCELL Technologies, Vancouver, BC, CA). This stimulus was selected to account for heightened diffusion

gradient and hypoxic exposure experienced by the bone marrow compartment (14). BMDM's were assessed for phagocytosis capacity per manufacturer recommendation (Phagocytosis Assay Kit, Cayman Chemical, Ann Arbor, MI, USA) and gene expression as previously described. Aged mice performed HU (n=5 per group) as described immediately prior to reloading received a single unilateral intramuscular injection (50 µL) of untreated or treated BMDM's ($2x10^6$ cells) to the triceps surae group. We and others have previously demonstrated the efficacy of this methodology (3, 15). Following injection, mice recovered until 7d for tissue collection and muscle force analysis (n=4).

Cell culture experiments. Murine C2C12 myoblast and myotubes (CRL-1772, ATCC, Manassas, VA, USA), NOR-10 fibroblasts (CCL-197, ATCC, Manassas, VA, USA), and Raw264.7 macrophages (TIB-71, ATCC, Manassas, VA, USA) were used for in vitro experiments. C2C12 cells were plated in 6-12 well dishes and utilized at ~30% confluence (cellular proliferation) or grown to confluence for ~6 days in growth medium (DMEM 4.5 g/dL, 10% FBS, 1% penicillin-streptomycin) then differentiated into myotubes for ~4 days in myotube differentiation medium (DMEM 4.5 g/dL, 2% HS, 1% penicillin-streptomycin). NOR-10 cells were plated in 8-chamber culture slides and utilized at ~50% confluence for ~6 days in growth medium. Raw264.7 macrophages were plated in T75 flasks, 6-well dishes, or 8-chamber culture slides and grown to confluence in growth medium with the same macrophage protocols as previously described. Cellular hypoxia was performed as previously described. In separate groups of Raw264.7 cells, conditioned culture media was generated for 48 hours following initial hypoxic stimulus by washing cells 3x in warm PBS, incubating in fresh growth medium followed by centrifugation (300xg, min) and stored for future use. C2C12 and NOR-10 cells were treated with Raw264.7 culture media for 24 hours to examine the secondary effects on cellular proliferation, differentiation, lipid droplet content, or wound healing time course during scratch assay. Cellular proliferation was assessed by incubating cells for 6 hours with 10 µM 5-bromo-2'-deoxyuridine (BrdU, B23151, Thermo Fisher). After washing, cells were fixed (4% PFA) and permeabilized (0.1% Triton X-100) then incubated for 10 min in 1N HCl (4ºC), 2N HCl (4ºC), and phosphate/citric acid buffer followed by 3x washes in permeabilization buffer. Cells were then incubated with 1:100 BrdU primary (B35130,

Invitrogen), 1:500 secondary (A11001, Invitrogen), and 1:10,000 DAPI (D3571, Invitrogen) antibodies. To assess myotube differentiation cells were washed, fixed, and permeabilized, blocked for 1 hour in 3% HS, then incubated with myosin heavy chain 4 conjugated antibodies (MF20, eBioscience, San Diego, CA, USA) for 1 hour (3% HS in PBS) followed by DAPI. Lipid droplet content was assessed following washes, fixing, and permeabilization by incubation with 2 µM BODIPY (D3992, Thermo Fisher) in PBS for 1hour followed by washing, 10 min incubation with DAPI, and washing. All cell conditions were imaged in triplicate (EVOS FL, Invitrogen) and averaged per well across condition replicates. Replicating cells were identified as the ratio of BrdU+/DAPI+. Myotube area as the field of view covered by functional myotubes (3+ nuclei) and fusion index as the number of nuclei contained within functional myotubes compared to total nuclei. Lipid droplet properties were determined using threshold analyzing and particle analysis. Scratch assay wound healing was quantified as relative distance grown/healed from initial wounding.

***Metabolomics*.** 90% methanol was added to samples to give a final concentration of 80%. Samples were incubated at -20ºC for 1 hour and centrifuged at 20,000 x g for 10 minutes at 4ºC, supernatants collected, and dried by vacuum. GC-MS analysis was performed with an Agilent 5977b GC-MS MSD-HES and Agilent 7693A automatic liquid sampler. Samples were suspended in 40 µL of 40 mg/mL O-methoxylamine hydrochloride (155405, MP Biomedicals, Santa Ana, CA, USA) in dry pyridine and incubated for one hour at 37 °C in a sand bath, then portions were transferred to autosampler tubes. 60 µL of N-methyl-N-trimethylsilyltrifluoracetamide (1% TMCS) was added and incubated for 30 min at 37 °C followed by vortexing and injection (1uL) into the gas chromatograph inlet in the split mode with the inlet temperature held at 250 °C. A 5:1 split ratio was used for analysis for the majority of metabolites. Saturated metabolites were analyzed at a 50:1 split ratio. The gas chromatograph had an initial temperature of 60 °C for one minute followed by a 10 °C/min ramp to 325 °C and a hold time of 10 minutes. A 30-meter Agilent Zorbax DB-5MS with 10 m Duraguard capillary column was employed for chromatographic separation. Helium was used as the carrier gas at a rate of 1 mL/min. Data was collected using MassHunter software (Agilent). Metabolites were identified and their peak area was

recorded using MassHunter Quant. This data was transferred to an Excel spread sheet. Metabolite identity was established using a combination of an in-house metabolite library developed using pure purchased standards, the NIST library and the Fiehn library. Data were median normalized, transformed (log2 fold change), and Pareto scaled using MetaboAnalyst/R software (v5.0) (16). Analysis including heatmap and PCA clustering, univariate analysis with 1.5 fold cutoff and p-adj <0.05, as well as KEGG and SMBPD pathway enrichment with ≥2 normalized enrichment score and ≤1.3 log2 p.

FACS. Gastrocnemius muscles dissected, minced, and incubated in digestion cocktail consisting of 100 mL of 5 mg/mL liberase (5401127001, Sigma Aldrich) and 25 mL of 10 U/mL DNAse (4716728001, Sigma Aldrich,) in 3 mL Ham's F12 media (11765054, ThermoFisher Scientific) with intermittent pipetting in a 37°C water bath for 45 min. Following digestion, homogenates were passed through 70 and 40 μm filters, and centrifuged at 1800 rpm for 10 min at 4°C. Supernatant was aspirated, and pellets resuspended in 1x red blood cell lysis buffer (420301, Biolegend, San Diego, CA, USA) for 10 min RT then neutralized with FACS buffer: F12 media with 10% Fetal bovine serum and 1% Penicillin-Streptomycin. Samples were spun at 1800 rpm for 10 min at 4°C for, supernatant aspirated, and pellets resuspended in FACS buffer. Cell pellets were incubated for FC Blocker CD16/CD32Monoclonal (Clone S17011E, Biolegend) for 5 min at 4°C and antibody mixture for 60 min in the dark at 4°C. Antibody mixture: BV450 anti-mouse CD31 (Clone 390, Biolegend), APC/Fire™ 750 anti-mouse CD45 (Clone 30-F11, Biolegend), PE anti-mouse F4/80 (Clone BM8, Biolegend), PerCP/Cyanine5.5 anti-mouse CD206 (Clone C068C2, Biolegend), PE/Cyanine7 anti-mouse Ly-6A/E (Sca-1) (Clone D7, Biolegend), APC a7-Integrin (Clone R2F2, AbLab, Vancouver, BC, CA). Samples were washed with FACS buffer spun at 1800 rpm 10 min at 4°C and resuspended in FACS buffer with DAPI. Immunophenotyping was performed using the BD Canto Cell Sorter equipped with BD FACSDiva software (v6.1.3). Data were analyzed using FlowJo™ v10.8 Software (BD Life Sciences) including manual compensation against single-color beads (A10497, ThermoFisher Scientific). Live cells (DAPI-) were gated from the CD31- population. Total macrophages (CD31-, CD45+, F4/80+), pro-inflammatory-

like (CD31-, CD45+, F4/80+, CD206-), satellite cells (CD31-, CD45-, Sca1-, α7integrin+) and fibro/adipogenic progenitor cells (CD31-, CD45-, Sca1+, α7integrin-) were gated and quantified calculated as percentage of total live cells or their parent populations.

References

1. Reidy PT, McKenzie AI, Mahmassani ZS, Petrocelli JJ, Nelson DB, Lindsay CC, et al. Aging impairs mouse skeletal muscle macrophage polarization and muscle-specific abundance during recovery from disuse. *Am J Physiol Endocrinol Metab.* 2019;317(1):E85-E98.
2. Fix DK, Ekiz HA, Petrocelli JJ, Mckenzie AM, Mahmassani ZS, O'Connell RM, et al. Disrupted macrophage metabolic reprogramming in aged soleus muscle during early recovery following disuse atrophy. *Aging Cell.* 2021;20(9):e13448.
3. Ferrara PJ, Yee EM, Petrocelli JJ, Fix DK, Hauser CT, de Hart NMMP, et al. Macrophage immunomodulation accelerates skeletal muscle functional recovery in aged mice following disuse atrophy. *J Appl Physiol (1985).* 2022;133(4):919-31.
4. Petrocelli JJ, Mahmassani ZS, Fix DK, Montgomery JA, Reidy PT, McKenzie AI, et al. Metformin and leucine increase satellite cells and collagen remodeling during disuse and recovery in aged muscle. *FASEB J.* 2021;35(9):e21862.
5. Gersh I, Hawkinson GE, and Rathbun EN. J. Cell Physiol; 1944:35-70.
6. Brooks SV, and Faulkner JA. Contractile properties of skeletal muscles from young, adult and aged mice. *J Physiol.* 1988;404:71-82.
7. Fennel ZJ, Bourrant PE, Kurian AS, Petrocelli JJ, de Hart NMMP, Yee EM, et al. Stem cell secretome treatment improves whole-body metabolism, reduces adiposity, and promotes skeletal muscle function in aged mice. *Aging Cell.* 2024;23(6):e14144.
8. Danckaert A, Trignol A, Le Loher G, Loubens S, Staels B, Duez H, et al. MuscleJ2: a rebuilding of MuscleJ with new features for high-content analysis of skeletal muscle immunofluorescence slides. *Skelet Muscle.* 2023;13(1):14.
9. Dungan CM, Peck BD, Walton RG, Huang Z, Bamman MM, Kern PA, et al. In vivo analysis of γH2AX+ cells in skeletal muscle from aged and obese humans. *FASEB J.* 2020;34(5):7018-35.
10. Zhang W, Karagiannidis I, Van Vliet ES, Yao R, Beswick EJ, and Zhou A. Granulocyte colony-stimulating factor promotes an aggressive phenotype of colon and breast cancer cells with biochemical changes investigated by single-cell Raman microspectroscopy and machine learning analysis. *Analyst.* 2021;146(20):6124-31.
11. Becker L, Lu CE, Montes-Mojarro IA, Layland SL, Khalil S, Nsair A, et al. Raman microspectroscopy identifies fibrotic tissues in collagen-related disorders via deconvoluted collagen type I spectra. *Acta Biomater.* 2023;162:278-91.
12. Unal M, Jung H, and Akkus O. Novel Raman Spectroscopic Biomarkers Indicate That Postyield Damage Denatures Bone's Collagen. *J Bone Miner Res.* 2016;31(5):1015-25.
13. Alix JJP, Plesia M, Dudgeon AP, Kendall CA, Hewamadduma C, Hadjivassiliou M, et al. Conformational fingerprinting with Raman spectroscopy reveals protein structure as a translational biomarker of muscle pathology. *Analyst.* 2024;149(9):2738-46.
14. Spencer JA, Ferraro F, Roussakis E, Klein A, Wu J, Runnels JM, et al. Direct measurement of local oxygen concentration in the bone marrow of live animals. *Nature.* 2014;508(7495):269-73.
15. Hsieh PL, Rybalko V, Baker AB, Suggs LJ, and Farrar RP. Recruitment and therapeutic application of macrophages in skeletal muscles after hind limb ischemia. *J Vasc Surg.* 2018;67(6):1908-20.e1.
16. Pang Z, Xu L, Viau C, Lu Y, Salavati R, Basu N, et al. MetaboAnalystR 4.0: a unified LC-MS workflow for global metabolomics. *Nat Commun.* 2024;15(1):3675.
