# Supplementary material for: Macrophage metabolic rewiring rejuvenates muscle Raman signatures and cellular remodeling during regrowth in aged mice
Source: JCI Insight. 2025 Sep 9;10(20):e194303. doi: 10.1172/jci.insight.194303 (PMC12581675; doi:10.1172/jci.insight.194303)

Uncropped HIF-1α blot from Supplemental Figure 2C.
>75kDa
Lanes 1-3, 7-9: control 1-3, 4-6.
Lanes 4-6, 10-12: hypoxia 1-3, 4-6.

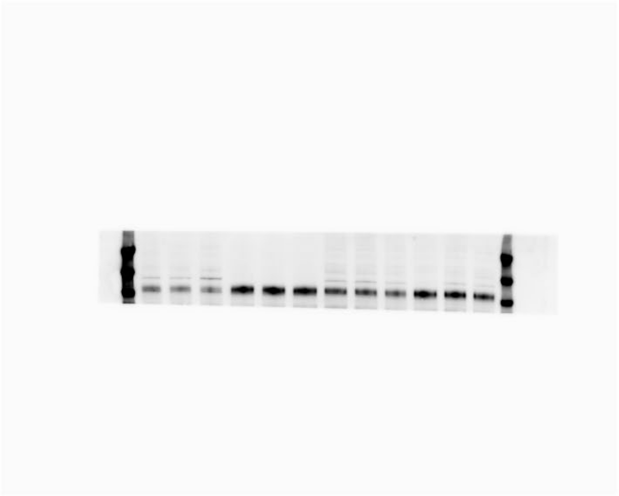

Uncropped p-4eBP1 blot from Supplemental Figure 2C
<30kDa
Lanes 1-3, 7-9: control 1-3, 4-6.
Lanes 4-6, 10-12: hypoxia 1-3, 4-6.

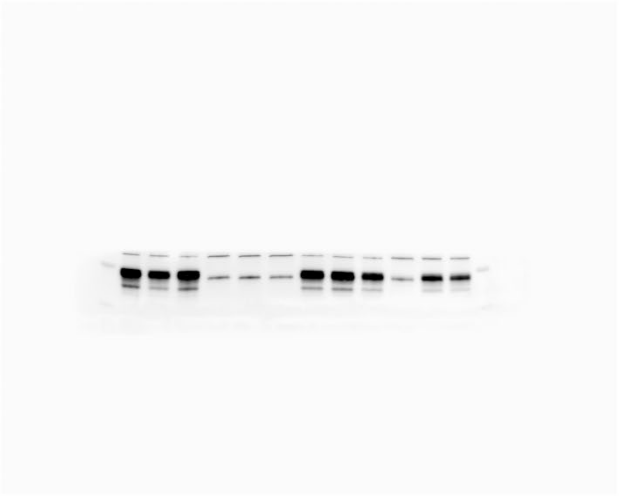

Uncropped Ponceau S stain from Supplemental Figure 2C
Whole-membrane used for above blots.

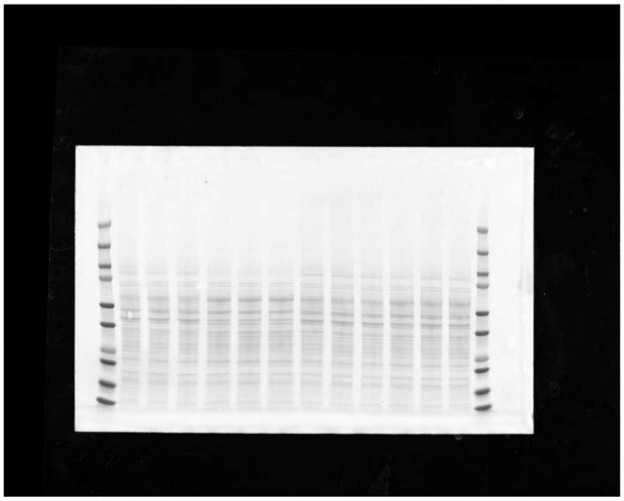

Supplement: Unedited blot and gel images [file jciinsight-10-194303-s241.pdf]
